# Cross-reactive serum and memory B-cell responses to spike protein in SARS-CoV-2 and endemic coronavirus infection

**DOI:** 10.1038/s41467-021-23074-3

**Published:** 2021-05-19

**Authors:** Ge Song, Wan-ting He, Sean Callaghan, Fabio Anzanello, Deli Huang, James Ricketts, Jonathan L. Torres, Nathan Beutler, Linghang Peng, Sirena Vargas, Jon Cassell, Mara Parren, Linlin Yang, Caroline Ignacio, Davey M. Smith, James E. Voss, David Nemazee, Andrew B. Ward, Thomas Rogers, Dennis R. Burton, Raiees Andrabi

**Affiliations:** 1 Department of Immunology and Microbiology, The Scripps Research Institute, La Jolla, CA USA; 2 IAVI Neutralizing Antibody Center, The Scripps Research Institute, La Jolla, CA USA; 3 Consortium for HIV/AIDS Vaccine Development (CHAVD), The Scripps Research Institute, La Jolla, CA USA; 4 Department of Integrative Structural and Computational Biology, The Scripps Research Institute, La Jolla, CA USA; 5 Division of Infectious Diseases, Department of Medicine, University of California, San Diego, La Jolla, CA USA; 6 Ragon Institute of Massachusetts General Hospital, Massachusetts Institute of Technology, and Harvard University, Cambridge, MA USA

**Keywords:** Antibodies, Viral infection, B cells, SARS-CoV-2

## Abstract

Pre-existing immunity to seasonal endemic coronaviruses could have profound consequences for antibody responses to SARS-CoV-2, induced from natural infection or vaccination. A first step to establish whether pre-existing responses can impact SARS-CoV-2 infection is to understand the nature and extent of cross-reactivity in humans to coronaviruses. Here we compare serum antibody and memory B cell responses to coronavirus spike proteins from pre-pandemic and SARS-CoV-2 convalescent donors using binding and functional assays. We show weak evidence of pre-existing SARS-CoV-2 cross-reactive serum antibodies in pre-pandemic donors. However, we find evidence of pre-existing cross-reactive memory B cells that are activated during SARS-CoV-2 infection. Monoclonal antibodies show varying degrees of cross-reactivity with betacoronaviruses, including SARS-CoV-1 and endemic coronaviruses. We identify one cross-reactive neutralizing antibody specific to the S2 subunit of the S protein. Our results suggest that pre-existing immunity to endemic coronaviruses should be considered in evaluating antibody responses to SARS-CoV-2.

## Abstract (web summary)

Pre-existing immune responses between antigenically related viruses can influence responses in viral infections or vaccinations. Here the authors assess and characterize the presence of antibody and memory B cell populations specific to SARS-CoV2 and endemic human coronaviruses.

## Introduction

Well-known examples of pre-existing immunity to viruses influencing antibody (Ab) responses to related viruses include original antigenic sin (OAS) in influenza virus infections and Ab-dependent enhancement (ADE) in flavivirus infections^1–3^. There is considerable interest in establishing whether Ab or T-cell responses to SARS-CoV-2, through infection or vaccination, might be impacted by pre-existing immunity to other coronaviruses, particularly the endemic coronaviruses (endemic HCoVs), namely the betacoronaviruses (β-HCoV), HCoV-HKU1 and HCoV-OC43, and the alphacoronaviruses (α-HCoV), HCoV-NL63 and HCoV-229E, which are responsible for non-severe infections such as common colds^4–8^. In principle, pre-existing immune perturbation effects could occur by the interaction of SARS-CoV-2 with cross-reactive circulating serum Abs or with B cells bearing cross-reactive B-cell receptors (BCRs) or T cells with cross-reactive T-cell receptors. Although a number of studies have reported on cross-reactive T cells and serum Abs^5,7,9–12^, we investigate here both Ab and BCR cross-reactivities.

## Results

Since individuals who have been infected with SARS-CoV-2 will generally also have been infected with endemic HCoVs, we chose to compare COVID-19 and pre-pandemic donors in terms of serum Abs and BCRs with specificity for the spike (S) protein (Supplementary Table 1 summarizes the demographic details of the human cohorts). The rationale was that the pre-pandemic donor cross-reactive responses in COVID-19 donors could have originated from endemic HCoV infection. Additinally, the COVID-19 cohort could reveal the effects of SARS-CoV-2 infection on cross-reactive responses.

**Fig. 1:**
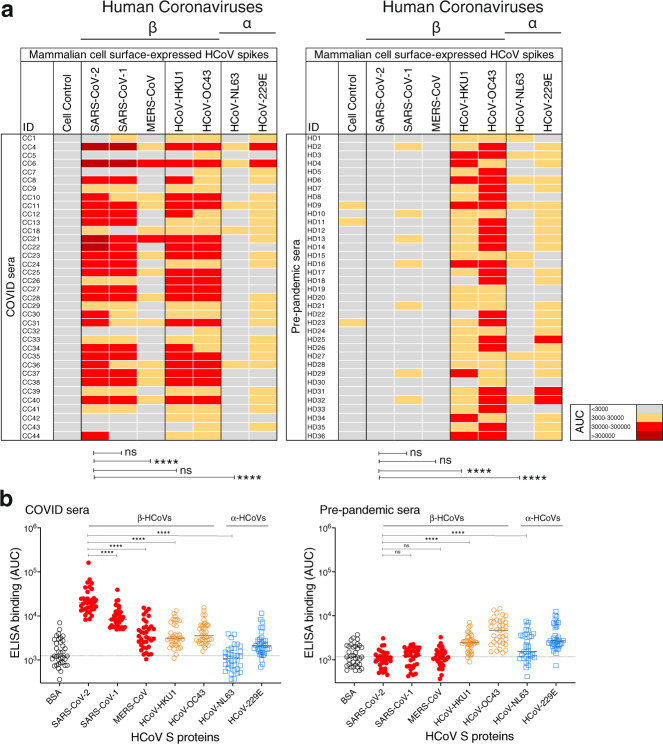
Reactivity of COVID and pre-pandemic human sera with cell surface-expressed human coronaviruses spikes and their soluble S-protein versions. **a** Heatmap showing cell-based flow cytometry binding (CELISA) of COVID and pre-pandemic donor sera with 293 T-cell surface-expressed full-length spike proteins from β-(SARS-CoV-2, SARS-CoV-1, MERS-CoV, HCoV-HKU1, HCoV-OC43) and α-(HCoV-NL63 and HCoV-229E) human coronaviruses (HCoVs). Sera were titrated (six dilutions–starting at 1:30 dilution) and the extent of binding to cell surface-expressed HCoVs was recorded by % positive cells, as detected by PE-conjugated anti-human-Fc secondary Ab using flow cytometry. Area-under-the-curve (AUC) was calculated for each binding titration curve and the antibody titer levels are color-coded as indicated in the key. The binding of sera to vector-only plasmid (non-spike) transfected 293 T cells served as a control for non-specific binding. **b** ELISA binding of COVID and pre-pandemic donor sera to soluble S-proteins from β-(SARS-CoV-2, SARS-CoV-1, MERS-CoV, HCoV-HKU1, HCoV-OC43) and α-(HCoV-NL63 and HCoV-229E) HCoVs. Serum dilutions (eight dilutions–starting at 1:30 dilution) were titrated against the S-proteins and the binding was detected as OD405 absorbance. AUC representing the extent of binding was calculated from binding curves of COVID (left) and pre-pandemic (right) sera with S-proteins and comparisons of antibody binding titers are shown. The binding of sera with each protein is shown as scatter dot plots with a line at median. Binding to BSA served as a control for non-specific binding by the sera. The serum-binding experiments were carried out in duplicate and repeated independently at least once for reproducibility. Statistical comparisons between two groups were performed using a Mann–Whitney two-tailed test, (****p < 0.0001; ns *p* > 0.05).

To assess serum Ab S-protein binding in the two cohorts, we used cell surface bound and recombinant soluble S-proteins. We employed both binding assays in parallel to assess any potential differences in the serum Ab-binding patterns that may result from the engineering and truncation of the soluble HCoV S protein relative to the membrane-bound protein. HIV envelope studies have revealed that the cell surface-expressed envelope trimers may more closely mimic infectious virion-associated envelope spikes, including in terms of native-like glycan compositions^13,14^. First, we developed and utilized a high-throughput flow cytometry-based cell surface spike-binding assay (cell-based enzyme-linked immunosorbent assay; CELISA). COVID-19 convalescent sera from 36 donors showed strong reactivity to the SARS-CoV-2 spike in the vast majority of infected donors (Fig. 1a, Supplementary Fig. 1), somewhat lower reactivity with the SARS-CoV-1 spike and much lower reactivity with the MERS-CoV spike in a pattern consistent with sequence conservation between the three viruses. COVID sera also exhibited strong cross-reactivity with endemic HCoV spikes, especially with the HCoV-HKU1 and HCoV-OC43 β-HCoVs (Fig. 1a). The α-HCoV-derived HCoV-NL63 spike was least reactive among the four endemic HCoVs. Next, we tested sera from a cohort of 36 HIV seropositive but otherwise healthy human donors whose samples were collected pre-pandemic. The sera showed minimal or no reactivity to SARS-CoV-2/CoV-1 and MERS-CoV spikes but showed strong binding to the endemic HCoV spikes, especially against the HCoV-HKU1 and HCoV-OC43 β-HCoVs (Fig. 1, Supplementary Fig. 1). The results suggest that the pre-pandemic sera, at least in our cohort, possess low levels of pre-existing SARS-CoV-2-circulating Abs.

To further investigate, we generated recombinant soluble S-proteins of all seven HCoVs using a general stabilization strategy described elsewhere^15–17^. ELISA showed a similar binding pattern of the COVID and pre-pandemic sera as the CELISA (Fig. 1b, Supplementary Fig. 1). The SARS-CoV-2 S-specific binding of COVID sera in the two assay formats (CELISA versus ELISA) correlated strongly (*r* = 0.92, *p* < 0.001) (Supplementary Fig. 2), the titers detected in ELISA being substantially lower overall. Differential sensitivity of the two assay formats may reflect an inherently greater sensitivity of flow cytometry (CELISA) compared with ELISA but also to the potential effects of engineering on the soluble HCoV S-proteins that may reduce nativity of some epitopes. We also tested the neutralization of the COVID sera with SARS-CoV-2 and the ID_50_ neutralization titers positively correlated with both binding assays (CELISA (*r* = 0.72, *p* < 0.0001), ELISA (*r* = 0.68, *p* < 0.0001)) (Supplementary Fig. 2). Overall, both CELISA and ELISA revealed binding Abs to all seven HCoV spikes in COVID sera but only to endemic HCoVs in the pre-pandemic sera.

To assess whether SARS-CoV-2 infection may impact serum Ab titers to endemic HCoVs, we compared Ab titers to endemic HCoV S-protein in sera from COVID and pre-pandemic cohorts. Higher CELISA Ab titers to endemic HCoV-HKU1 S-protein, but not for other HCoV spikes (HCoV-OC43, HCoV-NL63, and HCoV-229E) were observed in the COVID cohort compared with the pre-pandemic cohort (Supplementary Fig. 3). The result suggests that SARS-CoV-2 infection may boost titers to the related HCoV-HKU1 spike^18,19^. To further investigate, we divided individuals from the COVID cohort into two groups, one with the higher SARS-CoV-2 spike Ab titers (area-under-the-curve (AUC) > 85,000) and the other with lower titers (AUC < 85,000). Consistent with the above result, the COVID sera with higher SARS-CoV-2 titers showed significantly higher binding to HCoV-HKU1 and HCoV-OC43 S-proteins compared with the low titer group (Supplementary Fig. 3). The α-HCoVs HCoV-NL63 and HCoV-229E spike-binding Ab titers were comparable between the two groups and served as a control (Supplementary Fig. 3). As the two cohorts are not matched in terms of a number of parameters and are of limited size, any conclusions should be treated with caution. Nevertheless, it is noteworthy that SARS-CoV-2 infection is apparently associated with enhanced β-HCoVs S-protein Ab responses. A key question is whether the enhanced responses arise from de novo B-cell responses or from a recall response of B cells originally activated by an endemic HCoV virus infection.

**Fig. 2:**
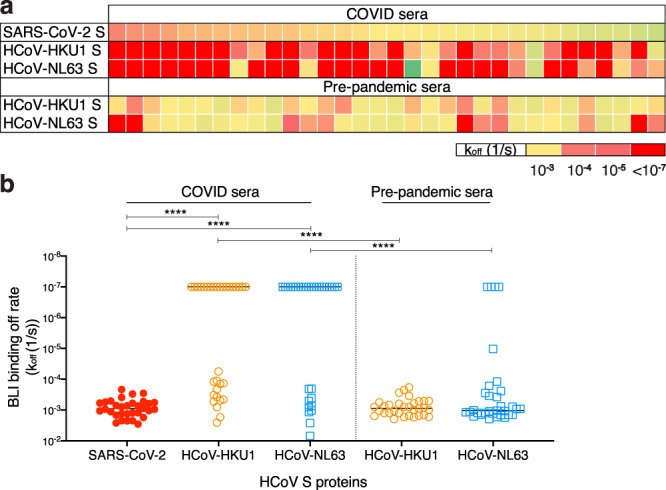
BioLayer interferometry binding of COVID and pre-pandemic serum antibodies to SARS-CoV-2 and endemic HCoV S-proteins. **a** Heatmap summarizing the apparent BLI binding off-rates (*k*_off_ (1/*s*)) of the COVID and pre-pandemic human serum antibodies to SARS-CoV-2 S and endemic β-HCoV, HCoV-HKU1 and α-HCoV, HCoV-NL63 S-proteins. Biotinylated HCoV S-proteins (100 nM) were captured on streptavidin biosensors to achieve binding of at least one response unit. The S-protein-immobilized biosensors were immersed in 1:40 serum dilution solution with serum antibodies as the analyte and the association (120 s; 180–300) and dissociation (240 s; 300–540) steps were conducted to detect the kinetics of antibody-protein interaction. *k*_off_ (1/*s*) dissociation rates for each antibody–antigen interaction are shown. **b** Off-rates for binding of serum antibodies from COVID donors and from pre-pandemic donors to SARS-CoV-2 S and endemic HCoV, HCoV-HKU1, and HCoV-NL63, S-proteins. Significantly lower dissociation off-rates are observed for COVID compared with pre-pandemic sera. Statistical comparisons between the two groups were performed using a Mann–Whitney two-tailed test, (****p < 0.0001).

We were encouraged to look more closely at the Abs involved by BioLayer Interferometry (BLI). Polyclonal serum antibodies were used as analytes with biotinylated S-proteins captured on streptavidin biosensors. As the concentrations of the S protein-specific polyclonal Abs in the sera are unknown, these measurements can provide an estimate of Ab dissociation off-rates (*k*_off_, which is Ab concentration-independent) but not binding constants^20^. Slower dissociation off-rates would indicate greater affinity maturation of antibodies with a given S protein^21^. It is important to note that the off-rates are likely associated with bivalent IgG binding (avidity) in the format used. Consistent with the notion of SARS-CoV-2 infection activating a recall of cross-reactive HCoV S-specific Abs, the COVID sera Abs exhibited significantly slower off-rates with HCoV-HKU1 and HCoV-NL63 S-proteins compared with pre-pandemic sera Abs (Fig. 2a–b, Supplementary Fig. 4).

Our hypothesis is that the increased affinity of cross-reactive Abs to endemic HCoVs in COVID donors arises from B-cell recall by SARS-CoV-2 heterologous boost. There is evidence from other studies that heterologous boosting can strongly drive B cell affinity maturation. For example, HIV infection has been shown to drive unusually high affinity maturation^21–26^. Presumably, the repeated exposure of B cells to a constantly diversifying epitope trains B cells to affinity mature with more diverse features within an epitope and increased somatic hypermutation (SHM). This idea is also supported by vaccination studies involving HIV antigens in engineered mice^27,28^. Another example is for human infections with two distinct flaviviruses. We previously showed that Zika virus infection in humans resulted in a strong recall of dengue-specific cross-reactive Ab responses from pre-existing dengue-specific memory B cells^29^. The cross-reactive recall B-cell responses had more mutations and higher apparent affinity for antigens from the earlier infection (dengue virus in this case) than against the antigens from the latter infection (Zika virus in this case).

**Fig. 3:**
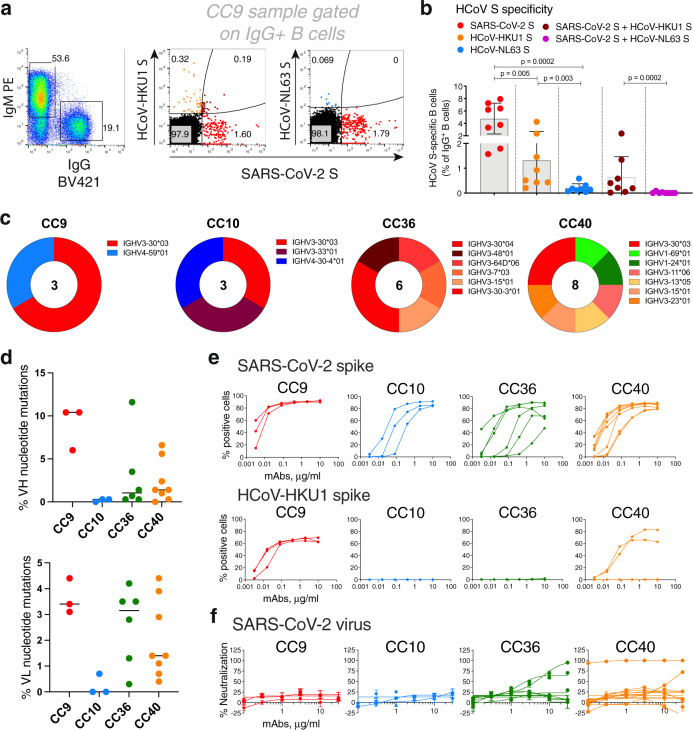
SARS-CoV-2 S and endemic HCoV S-protein-specific cross-reactive IgG+ memory B cells from COVID donors and isolation and characterization of mAbs. **a**–**b**. Flow cytometry analysis showing the single B-cell sorting strategy for COVID representative donor CC9 and frequencies of SARS-CoV-2 S and endemic β-HCoV, HCoV-HKU1 and α-HCoV, HCoV-NL63 S-protein-specific memory B cells in eight select COVID donors. The B cells were gated as SSL, CD3−, CD4−, CD8−, CD14−, IgD−CD19+, IgM−, IgG+. The frequencies of HCoV S-protein-specific IgG memory B cells were as follows; SARS-CoV-2 S (up to ~8%—range = ~1.6–8%), HCoV-HKU1 S (up to ~4.3%—range = ~0.2–4.3%), HCoV-NL63 S (up to ~0.6%–range = ~0.04–0.6%) protein single positive and SARS-CoV-2/HCoV-HKU1 S (up to ~2.4%–range = ~0.02–2.4%) and SARS-CoV-2/HCoV-NL63 S-protein (up to ~0.09%–range = ~0–0.09%) double positives. SARS-CoV-2-infected donors showed the presence of SARS-CoV-2/HCoV-HKU1 S-protein cross-reactive IgG memory B cells. Scatter dot plots show frequencies of S protein-specific B cells with a line at mean with SD. All differences between means with *p* values for each comparison are indicated. **p < 0.01; ***p < 0.001. A Mann–Whitney two-tailed test was used to compare the data groups. **c** Pie plots showing immunoglobulin heavy chain distribution of mAbs isolated from four COVID donors, CC9, CC10, CC36, and CC40. The majority of the mAbs were encoded by the IgVH3 immunoglobulin gene family. **d** Plots showing % nucleotide mutations in heavy (VH) and light (VL) chains of isolated mAbs across different individuals. The VH and VL mutations ranged from 0 to 11.6% and 0–4.4%, respectively, and are shown as scatter dot plots with a line at median. **e** CELISA-binding curves of isolated mAbs from four COVID donors with SARS-CoV-2 and HCoV-HKU1 spikes expressed on 293 T cells. Binding to HCoV spikes is recorded as % positive cells using a flow cytometry method. Five mAbs, three from the CC9 donor, and two from the CC40 donor show cross-reactive binding to SARS-CoV-2 and HCoV-HKU1 spikes. **f** Neutralization of SARS-CoV-2 by mAbs isolated from COVID donors. Four mAbs, two each from donors, CC36 and CC40, show neutralization of SARS-CoV-2. The neutralization experiments were performed in duplicate and repeated independently 1–2 times for reproducibility.

Having probed serum cross-reactivity between coronaviruses, we next investigated memory B cells in COVID individuals. We examined the reactivities of IgG+ memory B cells in eight select COVID donors (based on differential binding to HCoV spikes (Fig. 1) with SARS-CoV-2, HCoV-HKU1 (β-HCoV), and HCoV-NL63 (α-HCoV) S-proteins by flow cytometry. Up to ~8% SARS-CoV-2 S-protein, ~4.3% HCoV-HKU1 S-protein and ~0.6% for HCoV-NL63 S-protein-specific B cells were identified (Fig. 3a–b, Supplementary Fig. 5) in a frequency pattern consistent with serum Ab-binding titers.

**Fig. 4:**
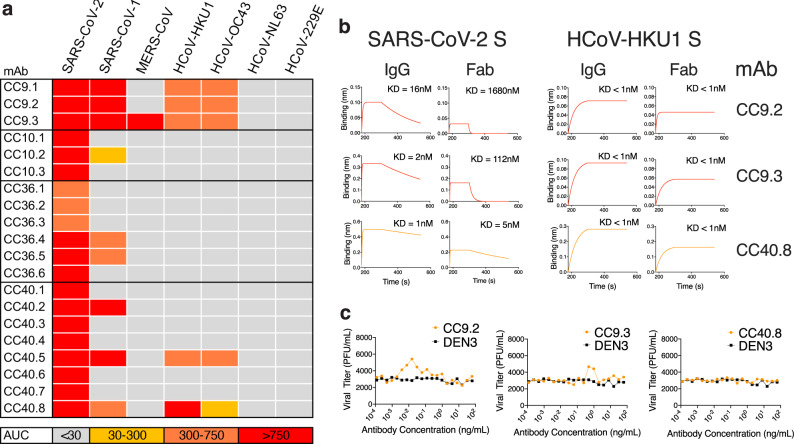
Binding and ADE of SARS-CoV-2/HCoV-HKU1 S-protein-specific cross-reactive mAbs. **a** Heatmap showing CELISA binding of COVID mAbs to seven HCoV spikes. Binding represented as area-under-the-curve (AUC) is derived from CELISA-binding titrations of mAbs with cell surface-expressed HCoV spikes and the extent of binding is color-coded. Five mAbs show cross-reactive binding across β-HCoV spikes. **b** BLI of SARS-CoV-2 and HCoV-HKU1 S-protein-specific cross-reactive mAbs. BLI binding of both IgG and Fab versions of three cross-reactive mAbs (CC9.2, CC9.3, and CC40.8) to SARS-CoV-2 and HCoV-HKU1 S-proteins was tested and the binding curves show association (120 s; 180–300) and dissociation rates (240 s; 300–540). BLI binding of antibody-S-protein combinations shows more stable binding (higher binding constants (KDs)) of cross-reactive mAbs HCoV-HKU1 compared to the SARS-CoV-2 S protein. **c** Antibody-dependent enhancement (ADE) activities of cross-reactive mAbs, CC9.2, CC9.3, and CC40.8 binding to SARS-CoV-2 live virus using FcγRIIa (K562) and FcγRIIb (Daudi)-expressing target cells. A dengue antibody, DEN3, was used as a control. Each data point in the curve is derived from the ADE experiment of mAbs with SARS-CoV-2 virus and shows virus titer obtained from technical replicates (*n* = 2); data representative of two independent experiments.

To probe the specificities of SARS-CoV-2/endemic HCoV cross-reactive Abs, we sorted single B cells for either SARS-CoV-2/HCoV-HKU-1 or SARS-CoV-2/HCoV-NL63 CoV S-protein double positivity. We isolated 20 S-protein-specific mAbs from four COVID donors, CC9 (*n* = 3), CC10 (*n* = 3), CC36 (*n* = 6), and CC40 (*n* = 8) (Fig. 3c, Supplementary Fig. 6) but only five mAbs, three from the CC9 donor and two from the CC40 donor, exhibited cross-reactive binding with HCoV-HKU1 spike (Fig. 3e). Two of the cross-reactive mAbs from the CC9 donor (CC9.1 and CC9.2) were clonally related. All five of the SARS-CoV-2/ HCoV-HKU-1 cross-reactive mAbs displayed binding to the genetically related β-HCoV, HCoV-OC43, spike but not to the α-HCoVs, HCoV-NL63, and HCoV-229E, spikes (Fig. 4a, Supplementary Fig. 7). Notably, one mAb (CC9.3) exhibited binding to five out of the seven HCoVs, including the MERS-CoV S-protein (Fig. 4a, Supplementary Fig. 7), suggesting targeting of a highly conserved epitope on β-HCoV spikes. One of the four SARS-CoV-2/HKU1-CoV S cross-reactive mAbs (CC40.8) showed weak cross-neutralization against SARS-CoV-2 and SARS-CoV-1 viruses (Supplementary Fig. 7). Except for CC9.3 mAb, all cross-reactive mAbs were encoded by VH3 heavy chain gene family (Supplementary Figs. 6 and 7) and possessed 5.6–10.4% (median = 6.6%) VH and 3.1–4.4% (median = 3.9%) VL nucleotide SHMs (Fig. 3d Supplementary Fig. 6).

In principle, the SARS-CoV-2/HCOV-HKU1 S cross-reactive memory B cells could be pre-existing in the COVID donors and show cross-reactivity with SARS-CoV-2 or originate from the SARS-CoV-2 infection and show cross-reactivity with HCoV-HKU1 S protein. The levels of SHM in the five cross-reactive mAbs listed above argue for the former explanation. To gain further insight, we conducted BLI-binding studies on the three cross-reactive mAbs, CC9.2, CC9.3, and CC40.8 (Fig. 4b). Both bivalent IgGs and monovalent Fabs showed enhanced binding affinity to HCoV-HKU1 S-protein compared with SARS-CoV-2 S-protein (Fig. 4b) again consistent with the notion that the Abs (BCRs) arise from a pre-existing HCoV-HKU1 S response. The serum and BCR data are then consistent. The data above suggests elevated serum levels of Abs to HCoV-HKU1 S-protein in COVID donors compared with pre-pandemic donors (Fig. 2a–b) is consistent with the notion that SARS-CoV-2 activates B cells expressing pre-existing HCoV-HKU1 S-protein-specific BCRs to secrete the corresponding Abs.

In general, it should be noted that although our study provides evidence for a recall of cross-reactive Abs upon SARS-CoV-2 infection, the most definitive demonstration of the origins of cross-reactive Ab responses would come from longitudinal human studies of donors before and after SARS-CoV-2 infection.

One mechanism by which pre-existing cross-reactive antibodies might influence the course of SARS-CoV-2 infection is ADE. Therefore, we investigated the potential ADE of the three cross-reactive Abs using a SARS-CoV-2 live virus assay (Fig. 4c). Of the three cross-reactive antibodies, CC9.3 mAb showed a marginal increase (twofold) in infection of SARS-CoV-2 virus in the FcγRIIa (K562) and FcγRIIb (Daudi) expressing target cells that can mediate ADE. Further in vivo assessment would be needed to determine whether this activity is associated with any meaningful physiological effects.

**Fig. 5:**
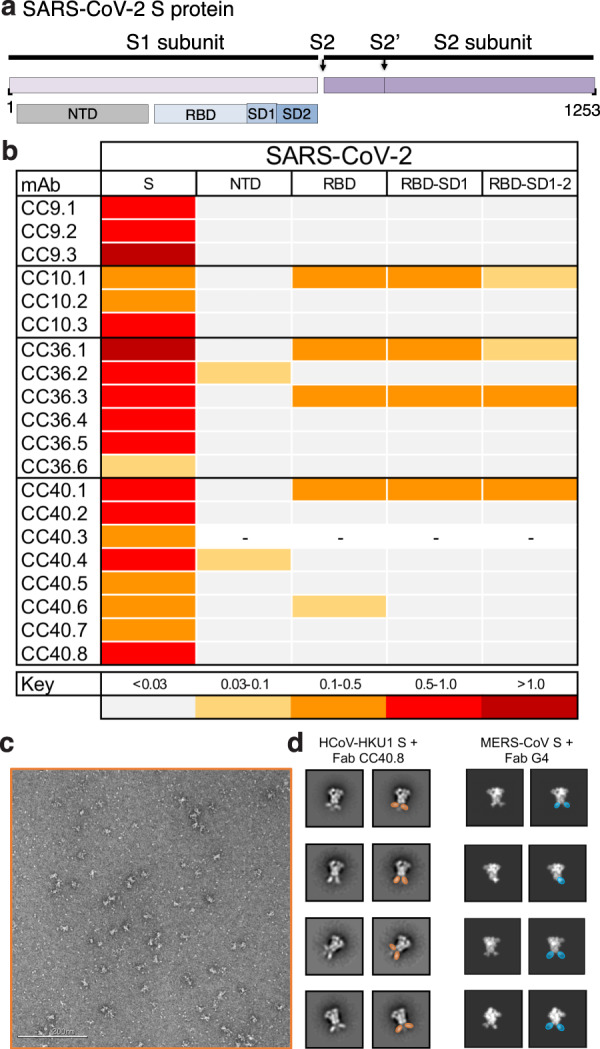
Epitope specificities of SARS-CoV-2 and endemic HCoV S-protein specific cross-reactive mAbs. **a**–**b** Organization of SARS-CoV-2 S protein subunits, domains, and subdomains (**a**). Epitope mapping of the mAbs binding to domains and subdomains of SARS-CoV-2 S-protein, NTD, RBD, RBD-SD1, and RBD-SD1-2 and heatmap showing BLI responses for each protein. The extent of binding responses is color-coded (**b**). Five mAbs were specific for RBD, two for NTD and the remaining mAbs displayed binding only to the whole S protein. **c**–**d** Negative stain electron microscopy of HCoV-HKU1 S-protein + Fab CC40.8 complex and comparison with MERS-CoV S + Fab G4 complex. **c** Raw micrograph of HCoV-HKU1 S in complex with Fab CC40.8. The Fab-HCoV-HKU1 S protein complexing was performed twice, and the data is representative of the two experiments. **d** Select reference-free 2D class averages with Fabs colored in orange for Fab CC40.8 and blue for Fab G4, which in 2D appear to bind a proximal epitope at the base of the trimer. 2D projections for MERS-CoV S-protein in complex with Fab G4 were generated in EMAN2 from PDB 5W9J.

To map the epitope specificities of the cross-reactive mAbs, we evaluated binding to a number of fragments of the S-protein (Fig. 5a–b). Notably, all five of the SARS-CoV-2/HKU1-CoV cross-reactive mAbs failed to bind any of the S1 subunit domains or subdomains, suggesting targeting to the more conserved S2 subunit. To identify the cross-reactive neutralizing epitope recognized by mAb CC40.8, we conducted structural studies of the Ab with the HKU1-CoV S protein. Using single-particle negative stain electron microscopy (nsEM) we observed that CC40.8 bound to the HCoV-HKU1 S trimer near the bottom of the S2 domain (Fig. 5c–d). The Fab density in the 2D class averages was blurry, suggesting binding to a flexible surface exposed peptide. The flexibility also precluded further 3D reconstruction.

Despite the requirement of double positivity in the B-cell sorting, 15/20 mAbs were largely specific for SARS-CoV-2. Again, like cross-reactive mAbs above, the vast majority of SARS-CoV-2-specific mAbs were encoded by VH3 gene family (Fig. 3c, Supplementary Fig. 6), consistent with other studies^30–37^. Overall, the SARS-CoV-2 spike antigenic surface can be recognized by various human VH-gene families, but there is a bias toward the VH3 gene-encoded antibodies and rational vaccine design strategies may take this feature into consideration. VH-germline gene-specific bias for antigenic shapes is common and has been previously reported for many pathogen surfaces^38–40^. Compared with the cross-reactive mAbs, the nucleotide SHM levels in SARS-CoV-2-specific mAbs were much lower (VH, 0–11.6% (median = 0.7%) VL, 0–4.2% (median = 1.3%)) (Fig. 3d Supplementary Fig. 6). Three of the 15 SARS-CoV-2 S-specific mAbs showed neutralization against SARS-CoV-2 virus, CC40.1 being the most potent (Fig. 3f, Supplementary Fig. 7). Some of the SARS-CoV-2-specific mAbs exhibited cross-reactive binding with SARS-CoV-1 S protein but none neutralized SARS-CoV-1 virus.

## Discussion

In conclusion, using a range of immune monitoring assays, we compared the serum and memory B-cell responses to the S-protein from all seven coronaviruses infecting humans in SARS-CoV-2 donors and in pre-pandemic donors. In sera from our pre-pandemic cohort, we found no evidence of pre-existing SARS-CoV-2 S-protein reactive antibodies that resulted from endemic HCoV infections, consistent with other studies^41,42^. A recent study has, however, reported the presence of SARS-CoV-2 S-protein reactive antibodies in a small fraction of pre-pandemic human sera from children and adolescents^11^. An in-depth examination for the presence of SARS-CoV-2 S-protein reactive antibodies in large pre-pandemic human cohorts is warranted to reliably determine the frequency of such antibodies. Notably, we observed serum levels of endemic HCoV S-protein antibodies were higher in SARS-CoV-2-experienced donors and memory B-cell studies suggested these likely arose from SARS-CoV-2 infection activating cross-reactive endemic HCoV S-protein-specific B cells. Cross-reactive mAbs largely target the more conserved S2 subunit on S-proteins and we identified a SARS-CoV-2 cross-neutralizing epitope that could facilitate vaccine design and Ab-based intervention strategies. Indeed, studies have shown targeting of conserved S2 subunit-neutralizing epitopes in SARS-CoV-2-infected donors and by SARS-CoV-1 nAbs that may potentially display activities against a broader range of human coronaviruses^43–46^. Overall, our study highlights the need to understand fully the nature of pre-existing endemic HCoV immunity in large and diverse human cohorts as vaccination of hundreds of millions of people against COVID-19 goes forward.

## Methods

### Human cohort information

Plasma and PBMCs from convalescent COVID patients were kindly provided through the “Collection of Biospecimens from Persons Under Investigation for 2019-Novel Coronavirus Infection to Understand Viral Shedding and Immune Response Study” UCSD IRB# 200236. Plasma from pre-pandemic donors was provided by Primary Infection Resource Consortium UCSD IRB# 140093 and 191008. These donors were from an HIV-1-positive healthy cohort of individuals with well-controlled HIV-1 and were on ARV. These pre-pandemic samples were collected from 18 April 2019 to 3 March 2020 before the spread of the pandemic in the United States. The protocol was approved by the UCSD Human Research Protection Program. COVID patient samples were collected based on COVID-19 diagnosis regardless of gender, race, ethnicity, disease severity, or other medical conditions. The age and the ethnicity variables were relatively evenly distributed across the two human cohorts (COVID and pre-pandemic samples). The gender distribution in the pre-pandemic cohort could not be controlled owing to the unavailability of the samples from female donors. The gender for individuals in the COVID cohort was evenly distributed. All human donors were assessed for medical decision-making capacity using a standardized, approved assessment, and voluntarily gave informed consent prior to being enrolled in the study. The summary of the demographic information of the COVID patients and pre-pandemic donors is listed in Supplementary Table 1.

### Plasmid construction for full-length and recombinant soluble proteins

To generate full-length human coronavirus plasmids, the spike genes were synthesized by GeneArt (Life Technologies). The SARS-CoV-1 (1255 amino acids; GenBank: AAP13567), SARS-CoV-2 (1273 amino acids; GenBank: MN908947), MERS-CoV (1353 amino acids; GenBank: APB87319.1), HCoV-HKU1 (1356 amino acids; GenBank: YP_173238.1), HCoV-OC43 (1361 amino acids; GenBank: AAX84792.1), HCoV-NL63 (1356 amino acids; GenBank: YP_003767.1) and HCoV-229E (1173 amino acids; GenBank: NP_073551.1) were cloned into the mammalian expression vector phCMV3 (Genlantis, USA) using PstI and BamH restriction sites. To express the soluble S ectodomain protein SARS-CoV-1 (residue 1–1190), SARS-CoV-2 (residue 1–1208), MERS-CoV (residue 1–1291), HCoV-HKU1 (residue 1–1295), HCoV-OC43 (residue 1–1300) and HCoV-NL63 (residue 1–1291), HCoV-229E (residue 1–1110), the corresponding DNA fragments were PCR amplified and constructed into vector phCMV3 using a Gibson assembly kit. To trimerize the soluble S-proteins and stabilize them in the prefusion state, we incorporated a C-terminal T4 fibritin trimerization motif in the C-terminal of each constructs and ﻿two consecutive proline substitutions in the S2 subunit^15–17^. To be specific, the K968/V969 in SARS-CoV-1, the K986/V987 in SARS-CoV-2, the V1060/L1061 in MERS-CoV, the A1071/L1072 in HCoV-HKU1, the A1078/L1079 in HCoV-OC43, the S1052/I1053 in HCoV-NL63 and the T871/I872 in HCoV-229E were replaced by proline residues. In addition, the S2 cleavage sites in each protein were replaced with a “GSAS” linker peptide. To facilitate the purification and biotin labeling of the soluble protein, the HRV-3C protease cleavage site, 6X HisTag, and AviTag spaced by GS-linkers were added to the C-terminus of the constructs, as needed. To express the SARS-CoV-2 N-terminal domain-NTD (residue 1–290), receptor-binding domain-RBD (residue 320–527), RBD-SD1 (residue 320–591), and RBD-SD1-2 (residue 320–681) subdomains, we amplified the DNA fragments by PCR reaction using the SARS-CoV-2 plasmid as template. All the DNA fragments were cloned into the vector phCMV3 (Genlantis, USA) in frame with the original secretion signal or the tissue plasminogen activator leader sequence. All the truncation proteins were fused to the C-terminal 6X HisTag, and AviTag spaced by GS-linkers to aid protein purification and biotinylation.

### Expression and purification of the proteins

To express the soluble S ectodomain proteins of each human coronavirus and the truncated versions, the plasmids were transfected into FreeStyle293F cells (Thermo Fisher). For general production, 350 µg plasmids were transfected into 1 L FreeStyle293F cells at the density of 1 million cells/mL. We mixed 350 µg plasmids with 16 mL transfectagro™ (Corning) and 1.8 mL 40 K polyethylenimine (PEI) (1 mg/mL) with 16 mL transfectagro™ in separate 50 mL conical tubes. We filtered the plasmid mixture with 0.22 μm Steriflip™ Sterile Disposable Vacuum Filter Units (MilliporeSigma™) before combining it with the PEI mixture. After gently mixing the two components, the combined solution rested at room temperature for 30 min and was poured into 1 L FreeStyle293F cell culture. To harvest the soluble proteins, the cell cultures were centrifuged at 2500 × *g* for 15 min on day 4 after transfection. The supernatants were filtered through the 0.22 μm membrane and stored in a glass bottle at 4°C before purification. The His-tagged proteins were purified with the HisPur Ni-NTA Resin (Thermo Fisher). To eliminate non-specific binding proteins, each column was washed with at least three bed volumes of wash buffer (25 mM Imidazole, pH 7.4). To elute the purified proteins from the column, we loaded 25 mL of the elution buffer (250 mM Imidazole, pH 7.4) at slow gravity speed (~4 sec/drop). Proteins without His tags were purified with GNL columns (Vector Labs). The bound proteins were washed with PBS and then eluted with 50 mL of 1 M Methyl α-d-mannopyranoside (Sigma M6882-500G) in PBS. By using Amicon tubes, we buffer exchanged the solution with PBS and concentrated the proteins. The proteins were further purified by size-exclusion chromatography using a Superdex 200 Increase 10/300 GL column (GE Healthcare). The selected fractions were pooled and concentrated again for further use.

### Biotinylation of proteins

Random biotinylation of S-proteins was conducted using EZ-Link NHS-PEG Solid-Phase Biotinylation Kit (Thermo Scientific #21440). In all, 10 µl dimethyl sulfoxide (DMSO) was added per tube for making concentrated biotin stock, 1 µl of which were diluted into 170 µl water before use. Coronavirus spike proteins were concentrated to 7–9 mg/ml using 100 K Amicon tubes in PBS, then aliquoted into 30 µl in PCR tubes. In all, 3 µl of the diluted biotin were added into each aliquot of concentrated protein and incubated on ice for 3 h. After reaction, buffer exchange for the protein was performed using PBS to remove excess biotin. BirA biotinylation of S-proteins was conducted using BirA biotin-protein ligase bulk reaction kit (Avidity). Coronavirus S-proteins with Avi-tags were concentrated to 7–9 mg/ml using 100 K Amicon tubes in tris-buffered saline (TBS), then aliquoted into 50 µl in PCR tubes. In all, 7.5 µl of BioB Mix, 7.5 µl of Biotin200, and 5 µl of BirA ligase (3 mg/ml) were added per tube. The mixture was incubated on ice for 3 h, followed by size-exclusion chromatography to segregate the biotinylated protein and the excess biotin. The extend of biotinylation was evaluated by BLI streptavidin biosensors.

### CELISA binding

The binding of serum antibodies or mAbs to human coronavirus spike proteins expressed on HEK293T cell surface was determined by flow cytometry, as described previously^47^. HEK293T cells were transfected with plasmids encoding full-length coronavirus spikes including SARS-CoV-1, SARS-CoV-2, MERS-CoV, HCoV-HKU1, HCoV-OC43, HCoV-NL63, and HCoV-229E. Transfected cells were incubated for 36–48 h at 37°C. Post incubation, cells were trypsinized to prepare a single-cell suspension and were distributed into 96-well plates. Serum samples were prepared as threefold serial titrations in fluorescence-activated cell sorting (FACS) buffer (1× PBS, 2% fetal bovine serum (FBS), 1 mM EDTA), starting at 1:30 dilution, six dilutions. In all, 50 μl/well of the diluted samples were added into the cells and incubated on ice for 1 h. The plates were washed twice in FACS buffer and stained with 50 μl/well of 1:200 dilution of R-phycoerythrin (PE)-conjugated mouse anti-human IgG Fc Ab (SouthernBiotech #9040-09) and 1:1000 dilution of Zombie-NIR viability dye (BioLegend) on ice in dark for 45 min. After another two washes, stained cells were analyzed using flow cytometry (BD Lyrics cytometers), and the binding data were generated by calculating the percent (%) PE-positive cells for antigen binding using FlowJo 10 software. CR3022, a SARS-CoV-1 and SARS-CoV-2 spike-binding ab, and dengue ab, DEN3, were used as positive and negative controls for the assay, respectively.

### ELISA binding

In all, 96-well half-area plates (Corning cat. #3690, Thermo Fisher Scientific) were coated overnight at 4°C with 2 µg/ml of mouse anti-His-tag Ab (Invitrogen cat. #MA1-21315-1MG, Thermo Fisher Scientific) in PBS. Plates were washed three times with PBS plus 0.05% Tween-20 (PBS-T) and blocked with 3% (wt/vol) bovine serum albumin (BSA) in PBS for 1 h. After removal of the blocking buffer, the plates were incubated with His-tagged spike proteins at a concentration of 5 µg/ml in 1% BSA plus PBS-T for 1.5 h at room temperature. After a washing step, perturbed and lotus serum samples were added in threefold serial dilutions in 1% BSA/PBS-T starting from 1:30 and 1:40 dilution, respectively, and incubated for 1.5 hr. CR3022 and DEN3 human antibodies were used as a positive and negative control, respectively, and added in 3-fold serial dilutions in 1% BSA/PBS-T starting at 10 µg/ml. After the washes, a secondary Ab conjugated with alkaline phosphatase (AffiniPure goat anti-human IgG Fc fragment specific, Jackson ImmunoResearch Laboratories cat. #109-055-008) diluted 1:1000 in 1% BSA/PBS-T, was added to each well. After 1 h of incubation, the plates were washed and developed using alkaline phosphatase substrate pNPP tablets (Sigma cat. #S0942-200TAB) dissolved in a staining buffer. The absorbance was measured after 8, 20, and 30 min, and was recorded at an optical density of 405 nm (OD405) using a VersaMax microplate reader (Molecular Devices), where data were collected using SoftMax software version 5.4. The wells without the addition of serum served as a background control.

### BLI binding

An Octet K2 system (ForteBio) was used for performing the binding experiments of the coronavirus spike proteins with serum samples. All serum samples were prepared in Octet buffer (PBS plus 0.1% Tween-20) as 1:40 dilution, random-biotinylated S-proteins were prepared at a concentration of 100 nM. The hydrated streptavidin biosensors (ForteBio) first captured the biotinylated spike proteins for 60 s, then transferred to Octet buffer for 60 s to remove unbound protein and provide the baseline. Then, they were immersed in diluted serum samples for 120 s to provide the association signal, followed by transferring into Octet buffer to test for disassociation signal for 240 s. The data generated were analyzed using the ForteBio Data Analysis software for correction and curve fitting, and for calculating the Ab dissociation rates (*k*_off_ values) or KD values for monoclonal antibodies.

### Flow cytometry B-cell profiling and mAb isolation with HCoV S-proteins

Flow cytometry of PBMC samples from convalescent human donors were conducted following methods described previously^32,48,49^. Frozen human PBMCs were re-suspended in 10 ml Rosewell Park Memorial Institute 1640 medium (Thermo Fisher Scientific, #11875085) pre-warmed to 37 °C containing 50% FBS. After centrifugation at 400 × *g* for 5 min, the cells were re-suspended in a 5 ml FACS buffer (PBS, 2% FBS, 2 mM EDTA) and counted. A mixture of fluorescently labeled antibodies to cell surface markers was prepared as 1:100 dilution that included antibodies specific for the T-cell markers CD3 (APC-Cy7, BD Pharmingen #557757), CD4 (APC-Cy7, Biolegend #317418), and CD8 (APC-Cy7, BD Pharmingen #557760); B-cell markers CD19 (PerCP-Cy5.5, Fisher Scientific #NC9963455), IgG (BV605, BD Pharmingen #563246) and IgM(PE); CD14 (APC-Cy7, BD Pharmingen #561384, clone M5E2). The cells were incubated with the Ab mixture for 15 min on ice in the dark. The SARS-CoV-2 S protein was conjugated to streptavidin-AF488 (Life Technologies #S11223), the HCoV-HKU1 S protein to streptavidin-BV421 (BD Pharmingen #563259) and the HCoV-NL63 S protein to streptavidin-AF647 (Life Technologies #S21374). Following conjugation, each S protein-probe was added to the Ab-cell mixture and incubated for 30 min on ice in the dark. FVS510 Live/Dead stain (Thermo Fisher Scientific, #L34966) in the FACS buffer (1:300) was added to the cells and incubated on ice in the dark for 15 min. The stained cells were washed with FACS buffer and re-suspended in 500 μl of FACS buffer/10-20 million cells, passed through a 70-μm mesh cap FACS tube (Fisher Scientific, #08-771-23) and sorted using a Beckman Coulter Astrios sorter, where memory B cells specific to S protein proteins were isolated. In brief, after the gating of lymphocytes (SSC-A vs. FSC-A) and singlets (FSC-H vs. FSC-A), live cells were identified by the negative FVS510 live/dead staining phenotype, then antigen-specific memory B cells were distinguished with sequential gating and defined as CD3−, CD4−, CD8−, CD14−, CD19+, IgM−, and IgG+. Subsequently, the S protein specific B cells were identified with the phenotype of AF488+BV421+(SARS-CoV-2/HCoV-HKU1 S protein double positive) or AF488+AF647+(SARS-CoV-2/HCoV-NL63 S protein double positive). Positive memory B cells were then sorted and collected at single-cell density in 96-well plates. Downstream single-cell IgG RT-PCR reactions were conducted using Superscript IV Reverse Transcriptase (Thermo Fisher, # 18090050), random hexamers (Gene Link # 26400003), Ig gene-specific primers, dNTP, Igepal, DTT and RNAseOUT (Thermo Fisher # 10777019). cDNA products were then used in nested PCR for heavy/light chain variable region amplification with HotStarTaq Plus DNA Polymerase (QIAGEN # 203643) and specific primer sets described previously^50,51^. The second round PCR exploited primer sets for adding on the overlapping region with the expression vector, followed by cloning of the amplified variable regions into vectors containing constant regions of IgG1, Ig Kappa, or Ig Lambda using Gibson assembly enzyme mix (New England Biolabs #E2621L) after confirming paired amplified product on 96-well E gel (ThermoFisher #G720801). Gibson assembly products were finally transformed into competent *Esherichia coli* cells and single colonies were picked for sequencing and analysis on IMGT V-Quest online tool (http://www.imgt.org) as well as downstream plasmid production for Ab expression.

### Neutralization assay

Under BSL2/3 conditions, MLV-gag/pol and MLV-CMV plasmids were co-transfected into HEK293T cells along with full-length or variously truncated SARS-CoV-1 and SARS-COV2 spike plasmids using Lipofectamine 2000 to produce single-round of infection competent pseudo-viruses. The medium was changed 16 h post transfection. The supernatant containing MLV-pseudotyped viral particles was collected 48 h post transfection, aliquoted and frozen at −80°C for neutralization assay. Pseudotyped viral neutralization assay was performed as previously described with minor modification (Modified from TZM-bl assay protocol^52^). In all, 293 T cells were plated in advance overnight with Dulbecco’s Modified Eagle medium +10% FBS + 1% Pen/Strep + 1% l-glutamine. Transfection was done with Opti-MEM transfection medium (Gibco, 31985) using Lipofectamine 2000. The medium was changed 12 h after transfection. Supernatants containing the viruses were harvested 48 h after transfection. (1) Neutralization assay for plasma. Plasma from COVID donors was heat-inactivated at 56 °C for 30 min. In sterile 96-well half-area plates, 25 μl of virus was immediately mixed with 25 μl of serially diluted (3×) plasma starting at 1:10 dilution and incubated for 1 h at 37°C to allow for Ab neutralization of the pseudotyped virus. In all, 10,000 HeLa-hACE2 cells/well (in 50 μl of media containing 20 μg/ml Dextran) were directly added to the Ab virus mixture. Plates were incubated at 37°C for 42–48 h. Following the infection, HeLa-hACE2 cells were lysed using 1× luciferase lysis buffer (25 mM Gly-Gly pH 7.8, 15 mM MgSO4, 4 mM EGTA, 1% Triton X-100). Luciferase intensity was then read on a Luminometer with luciferase substrate according to the manufacturer’s instructions (Promega, PR-E2620). (2) Neutralization assay for monoclonal antibodies. In 96-well half-area plates, 25 μl of virus was added to 25 μl of fivefold serially diluted mAb (starting concentration of 50 μg/ml) and incubated for 1 h before adding HeLa-ACE2 cell as mentioned above. Percentage of neutralization was calculated using the following equation: 100 × (1−(MFI of sample−average MFI of background)/average of MFI of probe alone−average MFI of background)).

### ADE assay

Ex vivo ADE quantification was measured using a focus reduction neutralization assay. Monoclonal antibodies were serially diluted in complete RPMI and incubated for 1 h at 37°C with SARS-CoV-2 strain USA-WA1/2020 (BEI Resources NR- 52281) [MOI = 0.01], in a BSL3 facility. Following the initial incubation, the mAb-virus complex was added in triplicate to 384-well plates seeded with 1E4 of K562 or Daudi cells and were incubated at 34°C for 24 h. In all, 20 µL of the supernatant was transferred to a 384-well plate seeded with 2E3 HeLa-ACE2 cells and incubated for an additional 24 h at 34°C. Plates were fixed with 25 µl of 8% formaldehyde for 1 h at 34°C. Plates were washed three times with 1× PBS 0.05% Tween-20 following fixation. 10 µL of human polyclonal sera diluted 1:500 in Perm/Wash Buffer (BD Biosciences) was added to the plate and incubated at RT for 2 h. The plates were then washed three times with 1× PBS 0.05% Tween-20 and stained with peroxidase goat anti-human Fab (Jackson Scientific, 109-035-006) diluted 1:2000 in Perm/wash buffer then incubated at RT for 2 h. The plates were then washed three times with 1× PBS 0.05% Tween-20. In all, 10 µL of Perm/Wash buffer was added to the plate then incubated for 15 min at RT. The Perm/Wash buffer was removed and 10 µL of TrueBlue peroxidase substrate was added. The plates were incubated for 30 min at RT then washed once with milli-Q water. The focus forming unit (FFU) per well was then quantified using a compound microscope. The PFU/mL of the monocyte plate supernatant was calculated and graphed using Prism 8 software.

### Negative stain electron microscopy

The HCoV-HKU1 S protein was incubated with a threefold molar excess of Fab CC40.8 for 30 mins at room temperature and diluted to 0.03 mg/ml in 1× TBS pH 7.4. In all, 3 μL of the diluted sample was deposited on a glow discharged copper mesh grid, blotted off, and stained for 55 s with 2% uranyl formate. Proper stain thickness and particle density was assessed on a FEI Morgagni (80 keV). The Leginon software^53^ was used to automate data collection on a FEI Tecnai Spirit (120 keV), paired an FEI Eagle 4k × 4k camera. The following parameters were used: 52,000× magnification, −1.5 μm defocus, a pixel size of 2.06 Å, and a dose of 25 e^−^/Å^2^. Micrographs were stored in the Appion database^54^, particles were picked using DogPicker^55^, and a particle stack of 256 pixels was made. RELION 3.0^56^ was used to generate the 2D class averages. The flexibility of the fab relative to the spike precluded 3D reconstruction.

### Statistical analysis

Statistical analysis was performed using Graph Pad Prism 8 for Mac, Graph Pad Software, San Diego, California, USA. Median AUC or reciprocal 50% binding (ID50) or neutralization (IC50) titers were compared using the non-parametric unpaired Mann–Whitney *U* test. The correlation between two groups was determined by Spearman rank test. Data were considered statistically significant at *p < 0.05, **p < 0.01, ***p < 0.001, and ****p < 0.0001.

### Reporting summary

Further information on research design is available in the Nature Research Reporting Summary linked to this article.

## Supplementary information

Supplementary Information

Reporting summary

## Data Availability

The authors declare that the data supporting the findings of this study are available within the paper and its supplementary information files or from the corresponding author upon reasonable request. Ab sequences have been deposited in GenBank under accession numbers MW426536-MW426544, MW532169-MW532198. Ab plasmids are available from Dennis Burton under an MTA from The Scripps Research Institute.
