# Supplementary material for: Cross-reactive serum and memory B-cell responses to spike protein in SARS-CoV-2 and endemic coronavirus infection
Source: Nat Commun. 2021 May 19;12:2938. doi: 10.1038/s41467-021-23074-3 (PMC8134462; doi:10.1038/s41467-021-23074-3)
Supplement: Supplementary file 1 — Supplementary Information [file 41467_2021_23074_MOESM1_ESM.pdf]

	COVID donor (n = 36)	Pre-pandemic donors (n = 36)
**Age** (years)	20–72 (median = 48)	23–79 (median = 43)
**Gender**		
Male	50% (18/36)	83% (30/36)
Female	50% (18/36)	17% (6/36)
**Race/Ethnicity**		
White, non-Hispanic	78% (28/36)	56% (20/36)
Hispanic	11% (4/36)	33% (12/36)
Black, non-Hispanic	0% (0/36)	8% (3/36)
Asian, non-Hispanic	5.5% (2/36)	3% (1/36)
Unknown	5.5% (2/36)	0% (0/36)
SARS-CoV-2 PCR Positivity	81% (29/36)	N/A
Lateral Flow Positivity	90% (25/36)	N/A
**Disease Severity**		
Mild	58% (21/36)	N/A
Mild to Moderate	5.5% (2/36)	N/A
Moderate	17% (6/36)	N/A
Moderate to Severe	11% (4/36)	N/A
Severe	5.5% (2/36)	N/A
Critical	3% (1/36)	N/A
**Symptoms**		
Cough	78% (28/36)	N/A
Fever	58% (21/36)	N/A
Fatigue	28% (10/36)	N/A
Anosmia	25% (9/36)	N/A
Dyspnea	25% (9/36)	N/A
Diarrhea	11% (4/36)	N/A
Days Post Symptom Onset at Collection	6–67 (median = 30)	N/A

Supplementary Table 1. Demographic information of COVID-19 and pre-pandemic HIV seropositive healthy human cohorts.

CELISA with cell surface expressed HCoV spikes — COVID sera

ID	Cell Control	SARS-CoV-2	SARS-CoV-1	MERS-CoV	HCoV-HKU1	HCoV-OC43	HCoV-NL63	HCoV-229E
CC1	299	2978	3278	1105	8879	9606	2464	13105
CC4	1320	705222	328987	13401	67422	67813	10518	51491
CC5	694	954	793	346	1090	6000	417	2619
CC6	350	649980	464439	59281	293716	161788	10080	70705
CC7	723	313	444	346	1804	8232	382	5654
CC8	637	42843	36534	619	47116	23031	1198	6897
CC9	686	22613	11752	927	15486	24412	934	2809
CC10	883	104842	28785	5970	36369	40470	1027	4684
CC11	277	91112	84670	10231	94022	61215	4145	13886
CC12	740	119327	57913	1091	46581	24631	760	3205
CC13	282	109787	120135	801	24664	23339	493	4044
CC18	347	14514	2625	4230	17350	16811	9011	7997
CC21	1069	484496	286635	37972	226279	109314	450	9353
CC22	467	375177	213577	1280	65423	30034	1785	1707
CC23	907	84101	41374	16920	40069	56723	332	12219
CC24	791	95600	32440	2264	18722	14736	1454	4989
CC25	880	278541	107803	21561	43201	46835	443	1996
CC26	736	16017	16018	1268	49955	67174	514	2766
CC27	486	156764	102058	2994	39868	38888	1144	1169
CC28	705	93624	81401	7150	48019	82962	467	4493
CC29	648	6507	4512	830	6065	11720	701	3955
CC30	348	69040	27867	1068	16304	21368	624	4968
CC31	700	53256	13030	6114	33668	76601	1046	6212
CC32	913	304	1392	995	1115	9196	677	2769
CC33	1119	24566	6435	739	13250	13191	541	3962
CC34	826	141541	36798	226	39114	29092	837	10813
CC35	1118	111268	32212	840	43035	43881	647	3722
CC36	1408	85078	21281	4723	48490	37109	10421	17391
CC37	762	253315	125133	15535	232063	162187	565	2239
CC38	1194	196105	44096	17283	44873	52371	1295	1538
CC39	1296	7664	3514	722	10233	12308	1031	3790
CC40	973	91930	77704	3611	158751	153295	528	4813
CC41	841	12500	4486	1219	9603	14253	887	3567
CC42	740	535	1324	783	10147	3778	577	2327
CC43	768	707	1415	436	2645	5853	1747	9571
CC44	1012	38583	2377	656	15036	17062	584	2028

CELISA with cell surface expressed HCoV spikes — Pre-pandemic sera

ID	Cell Control	SARS-CoV-2	SARS-CoV-1	MERS-CoV	HCoV-HKU1	HCoV-OC43	HCoV-NL63	HCoV-229E
HD1	616	496	1584	1101	6932	29467	11746	1804
HD2	1361	1124	3544	978	15061	69238	1789	7275
HD3	815	1074	1271	1279	74613	57110	3486	21039
HD4	269	725	1524	1177	39892	25081	1927	3753
HD5	675	818	1415	1322	5411	115314	1556	1025
HD6	485	1397	1721	926	128554	57957	17359	15080
HD7	1338	969	1858	1027	25598	39395	1011	3665
HD8	521	994	840	982	5081	37060	845	978
HD9	4148	775	745	831	64093	59791	4685	7404
HD10	516	801	14667	695	23286	7811	1643	6929
HD11	3742	557	885	1250	4615	78876	2045	4957
HD12	336	1043	1559	1010	9100	43373	1816	20308
HD13	651	413	3063	1978	12387	82850	946	4990
HD14	742	562	1445	1181	15620	84497	1894	19284
HD15	689	294	1729	677	5628	28740	5508	1881
HD16	1373	219	3089	887	46502	54273	3148	1677
HD17	825	438	2122	1468	17810	131424	2674	12838
HD18	350	1183	1711	1325	1697	63696	1596	25949
HD19	1023	988	1068	1336	13746	22750	318	1923
HD20	597	391	1195	1660	3261	16872	331	2584
HD21	356	764	3470	1245	12933	15104	334	8715
HD22	897	734	1472	1130	1110	30532	411	14658
HD23	4266	368	825	855	3129	59672	1652	3028
HD24	1835	774	631	1197	5735	19917	728	24232
HD25	737	834	764	956	4088	108933	1151	35542
HD26	940	613	644	1228	3261	41555	635	3675
HD27	543	1586	799	1258	14921	3884	3671	5657
HD28	503	340	847	1064	7820	12351	564	5494
HD29	677	1468	4365	972	36791	9366	762	4259
HD30	413	608	792	374	1207	12972	651	1882
HD31	352	1305	2659	916	4278	70517	430	30546
HD32	578	593	4717	1148	14668	60096	3761	45526
HD33	481	553	986	1331	27195	63364	1051	1802
HD34	785	363	1648	573	37968	16044	710	6197
HD35	581	686	1126	1119	11585	67111	2256	11935
HD36	1710	408	1734	1495	67911	30945	1044	19013

AUC	
<3000	
3000-30000	
30000-300000	
>300000	

Neutralization — COVID sera

ID	SARS-CoV-2	VSV-g
CC1	49	<20
CC4	2320	<20
CC5	<20	<20
CC6	1509	<20
CC7	<20	<20
CC8	<20	<20
CC9	38	<20
CC10	196	<20
CC11	85	<20
CC12	630	<20
CC13	38	<20
CC18	23	<20
CC21	1941	<20
CC22	406	<20
CC23	32	<20
CC24	24	<20
CC25	702	<20
CC26	<20	<20
CC27	51	<20
CC28	<20	<20
CC29	<20	<20
CC30	34	<20
CC31	<20	<20
CC32	<20	<20
CC33	<20	<20
CC34	97	<20
CC35	214	<20
CC36	115	<20
CC37	33	<20
CC38	72	<20
CC39	46	<20
CC40	151	<20
CC41	44	<20
CC42	44	<20
CC43	<20	<20
CC44	<20	<20

ELISA binding with HCoV S proteins — COVID sera

ID	BSA Control	SARS-CoV-2	SARS-CoV-1	MERS-CoV	HCoV-HKU1	HCoV-OC43	HCoV-NL63	HCoV-229E
CC1	756	14202	6105	1043	3163	4340	1077	2600
CC4	NA	196251	NA	NA	12837	28621	NA	NA
CC5	1811	10385	5538	1331	2157	2113	372	1499
CC6	748	161663	39274	12582	2144	2377	1263	11502
CC7	637	14359	8228	2585	1877	2335	732	1407
CC8	2073	16273	8127	5412	3187	4061	1803	1803
CC9	2750	17618	10164	7414	2962	2938	2300	3243
CC10	6998	41038	17352	14419	8346	5912	3815	7273
CC11	3542	19711	15298	10809	8359	6330	3932	8280
CC12	3522	24595	9543	4125	3738	2356	968	5382
CC13	361	15435	5545	1903	2336	3554	1561	1310
CC18	2936	14013	5026	2556	2871	4438	1156	2845
CC21	1043	67131	22748	4978	3045	5245	1374	1581
CC22	1228	57467	12184	1989	1093	2823	1741	2082
CC23	1226	20310	9702	4306	1762	2161	1207	996
CC24	805	25336	7839	5777	3108	6084	563	1786
CC25	4926	55363	22096	15347	13170	13116	2284	5394
CC26	3168	20018	10749	5051	9702	10447	822	2199
CC27	3908	44317	17794	5759	7290	10611	1856	4356
CC28	550	19048	11423	4257	3747	15376	956	2769
CC29	730	12291	7271	3034	2639	3239	627	1844
CC30	2369	16962	5720	3313	2825	2687	1004	1463
CC31	864	24759	12589	2612	3417	8119	640	2142
CC32	882	8491	5614	1354	2361	2830	761	2120
CC33	764	13293	5666	1872	1887	1896	551	2765
CC34	1029	24868	7037	3485	1813	1578	481	2304
CC35	1139	22905	8390	10063	1340	2610	739	1737
CC36	1055	21105	9438	1664	2260	2840	1283	1288
CC37	1202	35240	12773	2783	3098	2961	1329	798
CC38	2521	45589	16758	8275	3630	5083	1499	2959
CC39	3012	13864	8311	1326	4129	3425	2066	1329
CC40	1094	23015	11687	2903	6007	6202	3235	2520
CC41	1522	12060	6271	4767	11310	5672	493	2294
CC42	1454	8203	4947	2159	7776	2888	366	693
CC43	1522	10042	5073	1438	6464	6226	526	1748
CC44	1829	15727	5439	3085	8202	6939	785	1837

ELISA binding with HCoV S proteins — Pre-pandemic sera

ID	BSA Control	SARS-CoV-2	SARS-CoV-1	MERS-CoV	HCoV-HKU1	HCoV-OC43	HCoV-NL63	HCoV-229E
HD1	683	937	1513	1365	2378	2537	2093	1593
HD2	573	503	1896	1037	2601	3514	2168	3625
HD3	1257	1124	2030	1415	3604	14630	2121	5052
HD4	1078	530	2265	662	2900	2910	2699	1525
HD5	1219	628	1890	1668	2542	7154	2202	2038
HD6	1159	1228	1921	1943	5947	7592	1911	9366
HD7	2836	2144	1625	3260	2178	10653	2353	7034
HD8	2197	1428	1307	2080	2452	5664	1932	4850
HD9	687	451	1612	1090	893	5985	1601	4975
HD10	2007	1562	1089	1325	1124	2917	1681	6085
HD11	1796	1693	798	1656	1341	7400	1735	7693
HD12	566	630	232	612	1596	3371	1704	1352
HD13	885	995	1223	1662	2310	1725	842	2468
HD14	2298	768	1493	1462	4294	5700	583	2653
HD15	623	1122	1511	1010	1217	7995	817	2545
HD16	683	1423	1261	1068	3953	7169	1454	2340
HD17	762	912	1234	446	3262	9961	1404	2175
HD18	577	872	1043	617	1556	2153	1945	2400
HD19	2128	997	707	904	3269	2374	2039	2445
HD20	3719	1043	1872	2366	5565	4878	3748	1836
HD21	1008	510	425	1023	2824	9388	1770	1771
HD22	2940	3075	1272	1327	2674	2195	3065	3871
HD23	1499	837	488	784	2769	1569	2201	2038
HD24	1915	1200	971	1085	4259	11590	2532	1263
HD25	1944	1192	1920	1426	1361	4560	1316	4446
HD26	1153	1148	2185	1138	1939	1522	1095	2144
HD27	860	1484	234	1048	2619	2234	696	2874
HD28	782	906	480	1087	2481	6027	1012	2274
HD29	785	1217	450	736	2665	2567	665	3017
HD30	1046	1068	607	978	2237	1425	836	2943
HD31	2110	1309	702	368	1453	1651	426	1989
HD32	1451	848	663	1454	1498	7977	520	4357
HD33	727	554	727	835	2066	4798	502	2054
HD34	1144	604	756	1225	2329	3365	1429	2066
HD35	2033	1036	1606	1548	2196	3651	1845	1745
HD36	3122	2105	1664	1993	7031	13535	2465	1181

AUC	
<3000	
3000-30000	
30000-300000	
>300000	

Supplementary Figure 1. Binding of COVID and pre-pandemic sera with HCoV S proteins and neutralization of SARS-CoV-2 virus by COVID sera.

Binding of COVID and pre-pandemic sera to cell surface expressed HCoV spikes (CELISA) and soluble S proteins (ELISA). Binding is represented as area-under-the-curve (AUC) and was calculated for each binding titration curve. Binding of sera to non-spike vector only plasmid transfected 293T cells (cell control- CELISA) and to the BSA (ELISA) served as control for non-specific binding. SARS-CoV-2 virus specific ID50 neutralization titers of COVID sera. VSV-g virus served as control for the neutralization assay.

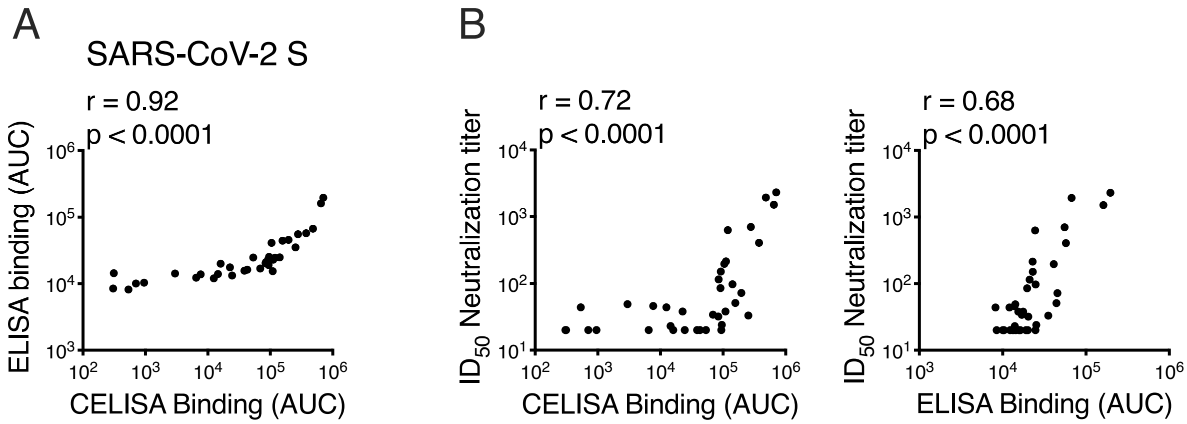

Supplementary Figure 2. Correlation of SARS-CoV-2 S binding with virus neutralization.

A. Correlation of COVID sera binding to 293T cell surface expressed SARS-CoV-2 spike (CELISA) with SARS-CoV-2 S protein ELISA binding. Binding titers were compared by nonparametric Spearman correlation two-tailed test with 95% confidence interval. The Spearman correlation coefficient (r) and the p-value is indicated.

B. Correlation of SARS-CoV-2 ID50 neutralization by COVID sera with CELISA and ELISA S protein binding. ID50 Neutralization titers of sera and CELISA (left) and ELISA (right) S protein binding titers were compared by nonparametric Spearman correlation two-tailed test with 95% confidence interval. The Spearman correlation coefficient (r) and the p-value is indicated.

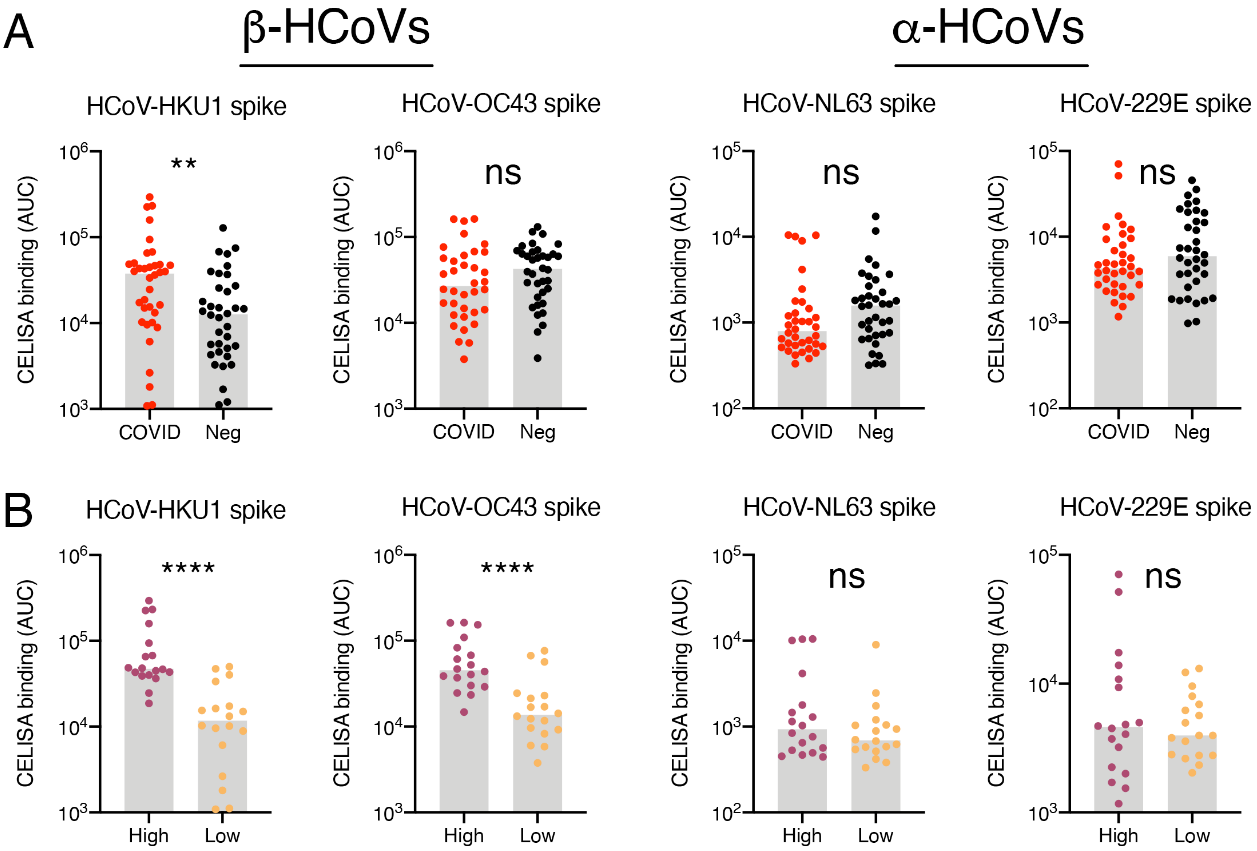

Supplementary Figure 3. Endemic HCoV S protein specific antibody titers in COVID and Pre-pandemic human sera.

A. Comparison of endemic HCoV S protein (β-HCoV: HCoV-HKU1 and HCoV-OC43 and α-HCoV: HCoV-NL63 and HCoV-229E) specific CELISA antibody binding titers between SARS-CoV-2 infected (COVID: n = 36) and non-infected healthy (Neg: n = 36) donors. COVID sera showed higher levels of antibody titers against β-HCoVs, HKU1-CoV and OC43-CoV compared to healthy sera but the antibody levels between the two groups were comparable for binding to α-HCoV S proteins, HCoV-NL63 and HCoV-229E. Statistical comparisons between two groups were performed using Mann-Whitney two tailed test, (**p <0.01; ns- p >0.05). p = 0.006 for COVID vs Neg sera for binding to HCoV-HKU1 spike.

B. Comparison of endemic HCoV S protein specific CELISA antibody binding titers in COVID donors, with SARS-CoV-2 S specific high (n = 18) and low (n = 18) antibody binding titers. The COVID donors with higher levels of SARS-CoV-2 S specific antibody titers display significantly higher binding with endemic β-HCoV, HCoV-HKU1 and HCoV-OC43 S proteins compared to individuals with lower SARS-CoV-2 S specific antibody titers. No significant difference for binding to α-HCoV S proteins, HCoV-NL63 and HCoV-229E between the two groups. Statistical comparisons between two groups were performed using Mann-Whitney two tailed test, (**p <0.01; ***p < 0.001, ****p < 0.0001; ns- p >0.05).

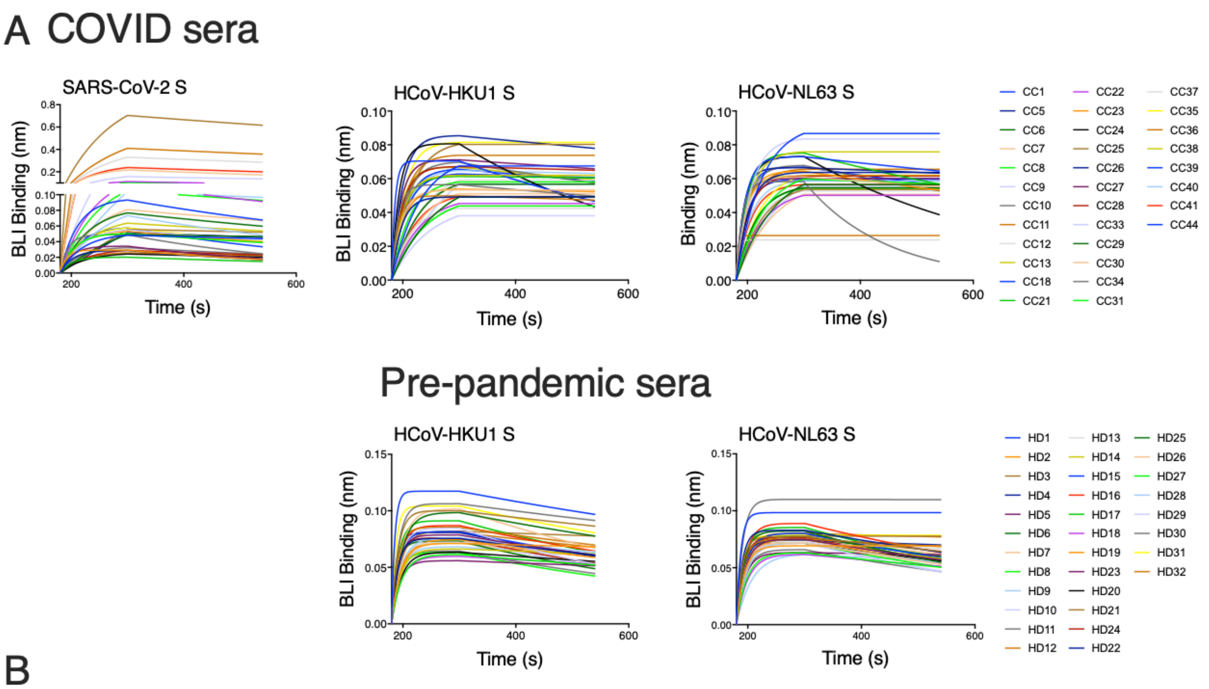

B

	COVID sera							Pre-pandemic sera			
	SARS-CoV-2 S		HCoV-HKU1 S		HCoV-NL63 S			HCoV-HKU1 S		HCoV-NL63 S	
	Response	k_{off}	Response	k_{off}	Response	k_{off}		Response	k_{off}	Response	k_{off}
CC9	0.044	2.17E-04	0.036	<1.0E-07	0.082	<1.0E-07	HD1	0.136	8.00E-04	0.114	<1.0E-07
CC30	0.052	2.87E-04	0.050	<1.0E-07	0.048	<1.0E-07	HD2	0.083	1.84E-04	0.082	<1.0E-07
CC37	0.100	3.02E-04	0.057	<1.0E-07	0.051	<1.0E-07	HD3	0.118	6.09E-04	0.094	7.51E-04
CC1	0.051	3.92E-04	0.069	<1.0E-07	0.083	<1.0E-07	HD4	0.091	9.06E-04	0.101	1.10E-03
CC36	0.107	5.11E-04	0.080	<1.0E-07	0.070	<1.0E-07	HD5	0.095	1.07E-03	0.091	1.16E-03
CC31	0.048	5.28E-04	0.063	<1.0E-07	0.056	<1.0E-07	HD6	0.119	9.86E-04	0.102	1.69E-03
CC6	0.700	5.54E-04	0.047	<1.0E-07	0.053	<1.0E-07	HD7	0.122	1.73E-03	0.090	1.26E-03
CC21	0.412	5.71E-04	0.074	5.61E-05	0.092	1.17E-03	HD8	0.073	6.85E-04	0.079	9.34E-04
CC38	0.163	5.95E-04	0.078	4.90E-04	0.072	<1.0E-07	HD9	0.091	8.02E-04	0.088	1.19E-03
CC22	0.334	6.03E-04	0.043	<1.0E-07	0.050	<1.0E-07	HD10	0.104	1.97E-03	0.091	1.76E-03
CC41	0.029	6.20E-04	0.048	<1.0E-07	0.064	<1.0E-07	HD11	0.120	6.27E-04	0.127	1.04E-05
CC23	0.070	7.11E-04	0.065	1.20E-04	0.078	7.95E-04	HD12	0.104	8.54E-04	0.091	3.82E-04
CC29	0.025	7.28E-04	0.053	<1.0E-07	0.052	<1.0E-07	HD13	0.086	5.07E-04	0.085	2.81E-04
CC25	0.257	7.37E-04	0.079	<1.0E-07	0.049	<1.0E-07	HD14	0.080	2.73E-04	0.092	1.05E-03
CC35	0.086	7.83E-04	0.080	<1.0E-07	0.057	<1.0E-07	HD15	0.098	1.24E-03	0.096	1.29E-03
CC27	0.121	8.76E-04	0.083	3.24E-04	0.071	<1.0E-07	HD16	0.107	1.26E-03	0.110	1.41E-03
CC33	0.033	9.46E-04	0.051	<1.0E-07	0.053	<1.0E-07	HD17	0.111	1.53E-03	0.106	1.63E-03
CC10	0.239	9.53E-04	0.071	5.34E-04	0.075	6.93E-03	HD18	0.072	5.11E-04	0.074	7.80E-04
CC34	0.085	1.05E-03	0.085	8.05E-04	0.084	1.04E-03	HD19	0.088	9.96E-04	0.091	1.43E-03
CC11	0.059	1.07E-03	0.062	2.13E-04	0.029	<1.0E-07	HD20	0.077	5.66E-04	0.089	1.01E-03
CC13	0.061	1.13E-03	0.071	<1.0E-07	0.084	<1.0E-07	HD21	0.092	2.23E-04	0.083	<1.0E-07
CC28	0.062	1.23E-03	0.075	1.36E-04	0.069	<1.0E-07	HD22	0.097	1.26E-03	0.090	2.43E-04
CC39	0.038	1.24E-03	0.070	1.42E-04	0.068	<1.0E-07	HD23	0.067	3.49E-04	0.077	1.19E-04
CC26	0.025	1.32E-03	0.099	3.82E-04	0.079	2.07E-04	HD24	0.094	1.46E-03	0.094	1.24E-03
CC24	0.110	1.34E-03	0.104	2.58E-03	0.095	2.65E-03	HD25	0.090	1.69E-03	0.094	1.07E-03
CC40	0.066	1.47E-03	0.073	1.12E-04	0.064	<1.0E-07	HD26	0.084	7.87E-04	0.093	9.12E-04
CC12	0.076	1.75E-03	0.041	<1.0E-07	0.024	<1.0E-07	HD27	0.080	1.67E-03	0.090	1.46E-03
CC7	0.035	2.22E-03	0.065	<1.0E-07	0.075	4.09E-04	HD28	0.082	5.25E-04	0.070	3.52E-04
CC5	0.037	2.23E-03	0.057	<1.0E-07	0.070	<1.0E-07	HD29	0.092	1.57E-03	0.094	1.98E-03
CC44	0.066	2.26E-03	0.077	4.55E-04	0.081	7.35E-04	HD30	0.082	1.55E-03	0.083	1.44E-03
CC8	0.043	2.64E-03	0.044	<1.0E-07	0.064	2.03E-04	HD31	0.122	1.08E-03	0.093	<1.0E-07
CC18	0.062	2.86E-03	0.090	1.72E-03	0.087	5.12E-04	HD32	0.097	7.45E-04	0.095	1.64E-04

Supplementary Figure 4. A. BioLayer Interferometry (BLI) binding curves of COVID and pre-pandemic sera with SARS-CoV-2, HCoV-HKU1 and HCoV-NL63 S proteins.
B. Binding responses and antibody binding off-rates (k_{off}) of COVID/pre-pandemic sera with SARS-CoV-2, HCoV-HKU1 and HCoV-NL63 S proteins.

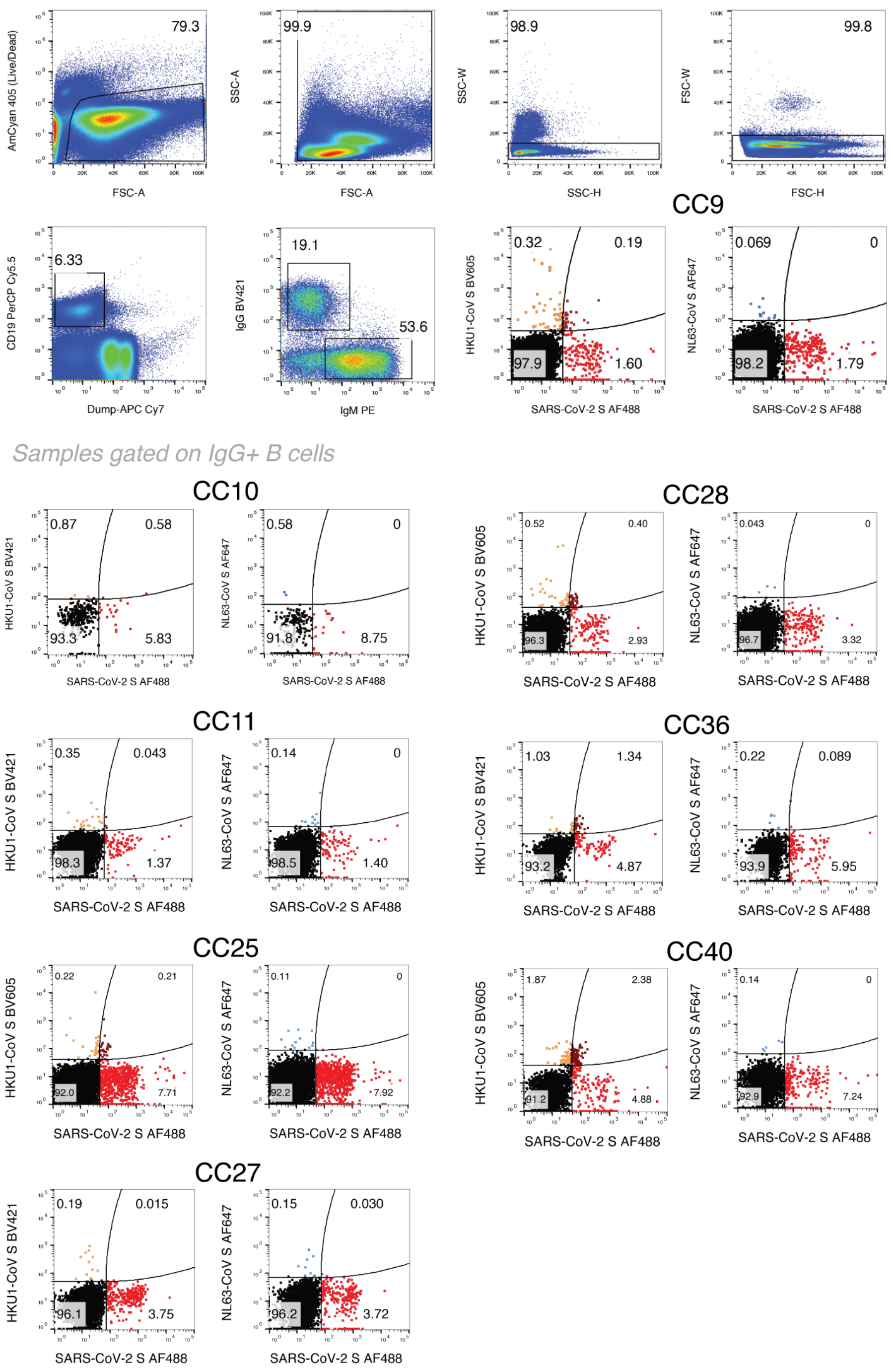

Supplementary Figure 5. Flow cytometry IgG+ memory B cell profiling of select COVID19 donors with SARS-CoV-2 S and endemic HCoV-HKU1/HCoV-NL63 S-protein probes.

Flow cytometry analysis of the 8 COVID19 donor IgG+ memory B cells specific to SARS-CoV-2 S and endemic β-HCoV, HCoV-HKU1 and α-HCoV, HCoV-NL63 S-protein. The B cells were gated as SSL, CD4-, CD8-, CD11C-, IgD-, IgM-, CD19+, IgG+. The frequencies of HCoV S-protein-specific IgG memory B cells are indicted for each individual. Flow cytometry profiling of the PBMC samples was performed in 3 independent sample

batches, as follows, (CC9, CC25, CC28 and CC40), (CC11, CC27 and CC36) and (CC10).

Donor ID	mAb	VH gene	% SHM (nt)	CDRH3	VL gene	% SHM (nt)	CDRL3
CC9	**CC9.1**	IGHV3-30*03	10.4	CVKGASLGDNW	IGKV2-30*01	3.1	CMQGTHWPYTF
	**CC9.2**	IGHV3-30*03	10.4	CVKGASLGDNW	IGKV2-30*01	3.4	CMQGTHWPYTF
	**CC9.3**	IGHV4-59*01	6.0	CARETRWNWLDSW	IGKV4-1*01	4.4	CQQYFNTPWTF
CC10	**CC10.1**	IGHV4-30-4*01	0.3	CARGKSPTVVTPWFDYW	IGLV1-40*01	0.0	CQSYDSSLSALWVF
	**CC10.2**	IGHV3-30*03	0.3	CAKDQSEGYGENYMDVW	IGLV3-25*03	0.7	CQSADSSDTWVF
	**CC10.3**	IGHV3-33*01	0.0	CAREGIVGATTGFDYW	IGLV3-10*01	0.0	CYSTDSSGNPSF
CC36	**CC36.1**	IGHV3-48*01	11.6	CARDRTYCSSGSCYAYDFFYGMDVW	IGLV1-51*02	3.5	CGTSDSSLSAGGVF
	**CC36.2**	IGHV3-64D*06	1.4	CVKDWAVLIQMPFDYW	IGLV7-46*01	4.2	CLLSNSGARPVF
	**CC36.3**	IGHV3-7*03	0.3	CARSSTYYTGFDYW	IGLV6-57*02	0.3	CQSYDGSNHQVF
	**CC36.4**	IGHV3-30-3*01	0.3	CARGLGGNYYAVDYW	IGKV3-20*01	2.8	CQQQGTF
	**CC36.5**	IGHV3-30*04	3.5	CARPYSGSYREYFQHW	IGKV4-1*01	1.3	CQQYYSTPITF
	**CC36.6**	IGHV3-15*01	0.7	CSRSILLYYYASTGYHHELDYW	IGLV2-14*01	3.5	CSSYTIISTLGVF
CC40	**CC40.1**	IGHV3-11*06	2.4	CARDSALDERGYSSGWHPPHW	IGKV1-5*03	2.9	CQQYNSYPWTF
	**CC40.2**	IGHV1-69*01	1.0	CARVGDYDSSGYYSDYW	IGKV3-11*01	1.4	CQQRSNWPPAVTF
	**CC40.3**	IGHV3-13*05	0.0	CARGGGRYYDFWSGYYGDYYHYYMDVW	IGKV2-29*02	0.7	CMQGIHLQVGITF
	**CC40.4**	IGHV1-24*01	1.4	CATGLQAARRDYYYYGMDVW	IGKV3-11*01	1.4	CQQRSNWPLTF
	**CC40.5**	IGHV3-30*03	6.6	CAKGQPLDDIW	IGKV2-30*02	4.4	CLQGTYWPWTF
	**CC40.6**	IGHV3-15*01	0.3	CTTEEPGAGSLYYYYYMDVW	IGKV3-11*01	0.4	CQQRSNWPRTF
	**CC40.7**	IGHV3-30*03	1.4	CAKDLAAWGPYCSSTNCYTGGMDVW	IGLV3-21*03	1.1	CQVWDSSSHWVF
	**CC40.8**	IGHV3-23*01	5.6	CAITMAPVVW	IGLV3-10*01	3.9	CYSTDSSGNHAVF

Supplementary Figure 6. Immunogenetic properties of the SARS-CoV-2 and HCoV-HKU1 S protein specific mAbs isolated from COVID donors, CC9, CC10, CC36 and CC40.

ELISA binding

mAb	SARS-CoV-2	SARS-CoV-1	MERS-CoV	HCoV-HKU1	HCoV-OC43	HCoV-NL63	HCoV-229E
CC9.1	30.2	15.8	2.2	8.6	9.6	2.6	2.7
CC9.2	29.3	15.9	2.2	7.9	8.4	2.6	2.8
CC9.3	31.0	25.4	30.4	11.8	11.0	2.5	2.8
CC10.1	27.5	2.2	2.3	2.2	1.9	2.7	2.8
CC10.2	14.6	2.3	2.2	2.2	1.8	2.7	2.8
CC10.3	30.8	2.5	2.8	2.5	2.0	3.0	3.2
CC36.1	30.1	2.0	2.2	2.5	1.8	2.6	2.6
CC36.2	29.6	2.1	2.4	2.6	1.9	2.8	2.9
CC36.3	31.0	2.7	3.0	2.5	1.9	2.7	2.8
CC36.4	30.9	19.0	2.7	2.6	2.0	2.8	2.9
CC36.5	30.6	22.5	2.8	2.5	2.0	2.9	3.0
CC36.6	29.4	2.5	2.6	2.3	2.0	3.0	3.0
CC40.1	31.1	2.2	2.8	2.7	1.9	2.9	2.9
CC40.2	31.0	27.8	2.5	2.4	1.8	2.6	2.7
CC40.3	3.3	3.4	-	1.4	-	-	-
CC40.4	30.8	2.1	2.3	2.4	1.8	2.5	2.6
CC40.5	2.6	2.1	2.1	3.8	2.7	2.5	2.6
CC40.6	6.1	2.1	2.1	2.4	1.8	2.5	2.5
CC40.7	2.4	2.1	2.0	2.5	1.8	2.5	2.6
CC40.8	30.5	24.3	2.7	27.5	4.9	2.5	2.6

AUC	<3	3-10.	10-25.	>25

BLI binding

mAb	SARS-CoV-2	SARS-CoV-1	MERS-CoV	HCoV-HKU1	HCoV-OC43	HCoV-NL63	HCoV-229E
CC9.1	0.79	0.68	0.02	0.13	0.05	0.01	0.02
CC9.2	0.53	0.42	0.00	0.10	0.03	0.00	0.02
CC9.3	1.13	0.80	0.78	0.18	0.08	0.02	0.03
CC10.1	0.39	0.00	0.01	0.00	0.00	0.00	0.01
CC10.2	0.29	0.00	0.00	0.00	0.00	0.00	0.01
CC10.3	0.53	0.00	0.00	0.00	0.00	0.00	0.00
CC36.1	1.10	0.00	0.03	0.00	0.00	0.00	0.00
CC36.2	0.76	0.00	0.01	0.00	0.00	0.00	0.00
CC36.3	0.74	0.05	0.00	0.00	0.00	0.00	0.00
CC36.4	0.77	0.48	0.01	0.00	0.00	0.01	0.00
CC36.5	0.77	0.55	0.02	0.00	0.00	0.01	0.00
CC36.6	0.08	0.00	0.00	0.00	0.00	0.00	0.00
CC40.1	0.95	0.00	0.02	0.00	0.00	0.00	0.00
CC40.2	0.68	0.45	0.01	0.00	0.00	0.01	0.01
CC40.3	0.42	0.39	0.01	0.00	0.00	0.01	0.01
CC40.4	0.66	0.00	0.01	0.00	0.00	0.00	0.00
CC40.5	0.23	0.10	0.00	0.10	0.04	0.00	0.02
CC40.6	0.46	0.00	0.01	0.00	0.00	0.00	0.00
CC40.7	0.36	0.00	0.00	0.00	0.00	0.00	0.00
CC40.8	0.71	0.49	0.02	0.10	0.01	0.01	0.03

Response	<0.03	0.03-0.1	0.1-0.5	0.5-1.0	>1.0

Neutralization

mAb	SARS-CoV-2	SARS-CoV-1
CC9.1	>30	>30
CC9.2	>30	>30
CC9.3	>30	>30
CC10.1	>30	>30
CC10.2	>30	>30
CC10.3	>30	>30
CC36.1	>30	>30
CC36.2	>30	>30
CC36.3	3.0	>30
CC36.4	>30	>30
CC36.5	>30	>30
CC36.6	4.4	>30
CC40.1	<0.1	>30
CC40.2	>30	>30
CC40.3	>30	>30
CC40.4	>30	>30
CC40.5	>30	>30
CC40.6	>30	>30
CC40.7	>30	>30
CC40.8	12.6	26.7

IC50 neutralization (ug/ml)

Supplementary Figure 7. ELISA binding, BLI binding and SARS-CoV-2/SARS-CoV-2 virus neutralization of mAbs isolated from 4 COVID donors, CC9, CC10, CC36 and CC40.
