# Supplementary material for: Cross-reactive serum and memory B-cell responses to spike protein in SARS-CoV-2 and endemic coronavirus infection
Source: Nat Commun. 2021 May 19;12:2938. doi: 10.1038/s41467-021-23074-3 (PMC8134462; doi:10.1038/s41467-021-23074-3)
Supplement: Supplementary file 2 — Reporting summary [file 41467_2021_23074_MOESM2_ESM.pdf]

nature research

Corresponding author(s): Raiees Andrabi/Dennis R. Burton

Last updated by author(s): 2021/01/25

Reporting Summary

Nature Research wishes to improve the reproducibility of the work that we publish. This form provides structure for consistency and transparency in reporting. For further information on Nature Research policies, see our Editorial Policies and the Editorial Policy Checklist.

Statistics

For all statistical analyses, confirm that the following items are present in the figure legend, table legend, main text, or Methods section.

n/a	Confirmed	
☐	☒	The exact sample size (n) for each experimental group/condition, given as a discrete number and unit of measurement
☐	☒	A statement on whether measurements were taken from distinct samples or whether the same sample was measured repeatedly
☐	☒	The statistical test(s) used AND whether they are one- or two-sided<br>*Only common tests should be described solely by name; describe more complex techniques in the Methods section.*
☐	☒	A description of all covariates tested
☐	☒	A description of any assumptions or corrections, such as tests of normality and adjustment for multiple comparisons
☐	☒	A full description of the statistical parameters including central tendency (e.g. means) or other basic estimates (e.g. regression coefficient) AND variation (e.g. standard deviation) or associated estimates of uncertainty (e.g. confidence intervals)
☐	☒	For null hypothesis testing, the test statistic (e.g. F, t, r) with confidence intervals, effect sizes, degrees of freedom and P value noted<br>*Give P values as exact values whenever suitable.*
☒	☐	For Bayesian analysis, information on the choice of priors and Markov chain Monte Carlo settings
☒	☐	For hierarchical and complex designs, identification of the appropriate level for tests and full reporting of outcomes
☒	☐	Estimates of effect sizes (e.g. Cohen's d, Pearson's r), indicating how they were calculated

Our web collection on statistics for biologists contains articles on many of the points above.

Software and code

Policy information about availability of computer code

Data collection	SoftMax Pro 5.4<br>Fortebio Data Acquisition 9.0<br>BD FACSuite v1.2.1<br>Beckman Coulter Summit V6.3.1<br>BioTek Gen5 2.09<br>Leginon software
Data analysis	GraphPad Prism 8<br>FlowJo (v10.7.1)<br>Fortebio Data Analysis 9.0<br>IMGT V-Quest online tool<br>RELION 3.0

For manuscripts utilizing custom algorithms or software that are central to the research but not yet described in published literature, software must be made available to editors and reviewers. We strongly encourage code deposition in a community repository (e.g. GitHub). See the Nature Research guidelines for submitting code & software for further information.

Data

Policy information about availability of data

All manuscripts must include a data availability statement. This statement should provide the following information, where applicable:

- Accession codes, unique identifiers, or web links for publicly available datasets
- A list of figures that have associated raw data
- A description of any restrictions on data availability

The authors declare that the data supporting the findings of this study are available within the paper and its supplementary information files or from the corresponding author upon reasonable request. Antibody sequences have been deposited in GenBank under accession numbers MW426536-MW426544 and MW532169-MW532198. Antibody plasmids are available from Dennis Burton under an MTA from The Scripps Research Institute.

Field-specific reporting

Please select the one below that is the best fit for your research. If you are not sure, read the appropriate sections before making your selection.

[x] Life sciences [ ] Behavioural & social sciences [ ] Ecological, evolutionary & environmental sciences

For a reference copy of the document with all sections, see nature.com/documents/nr-reporting-summary-flat.pdf

Life sciences study design

All studies must disclose on these points even when the disclosure is negative.

Sample size	The sample size was based on availability of the samples and patients.
Data exclusions	No data were excluded from the analyses.
Replication	For reproducibly, experimental assays were carried out in duplicates and repeated at least once for reproducibility, and all attempts at replication were successful.
Randomization	Randomization is not applicable in this study because the samples used for each experiments were grouped and data was obtained in the same batch in order to generate accurate comparison.
Blinding	The reported data in this study was based on quantitative measurements, including binding experiments and neutralization experiments etc. Therefore, blinding was not relevant given the fact that there is no subjective observations in this study.

Reporting for specific materials, systems and methods

We require information from authors about some types of materials, experimental systems and methods used in many studies. Here, indicate whether each material, system or method listed is relevant to your study. If you are not sure if a list item applies to your research, read the appropriate section before selecting a response.

Materials & experimental systems

n/a	Involved in the study	
[ ]	[x]	Antibodies
[ ]	[x]	Eukaryotic cell lines
[x]	[ ]	Palaeontology and archaeology
[x]	[ ]	Animals and other organisms
[ ]	[x]	Human research participants
[x]	[ ]	Clinical data
[x]	[ ]	Dual use research of concern

Methods

n/a	Involved in the study	
[x]	[ ]	ChIP-seq
[ ]	[x]	Flow cytometry
[x]	[ ]	MRI-based neuroimaging

Antibodies

Antibodies used	R-phycoerythrin (PE)-conjugated mouse anti-human IgG Fc antibody (SouthernBiotech #9040-09); AffiniPure goat anti-human IgG Fc fragment specific (Jackson ImmunoResearch Laboratories #109-055-008); mouse anti-His-tag antibody (Invitrogen #MA1-21315); anti-human CD3(APC Cy7, BD Pharmingen #557757), anti-human CD4(APC-Cy7, Biolegend #317418), anti-human CD8(APC-Cy7, BD Pharmingen #557760), anti-human CD14(APC-Cy7, BD Pharmingen #561384, clone M5E2), anti-human CD19(PerCP-Cy5.5, BioLegend #302230), anti-human IgG(BV605, BD Pharmingen #563246) and anti-human IgM(PE, BioLegend #314508); Peroxidase AffiniPure goat anti-human IgG F(ab')2 fragment specific (Jackson Scientific, 109-035-006)
Validation	All commercial antibodies have validation on manufacturer's website as follows: https://www.southernbiotech.com/?catno=9040-09&type=Monoclonal#&panel2-1

https://www.jacksonimmuno.com/catalog/products/109-055-008
https://www.thermofisher.com/antibody/product/6x-His-Tag-Antibody-clone-HIS-H8-Monoclonal/MA1-21315
https://www.bdbiosciences.com/us/reagents/research/antibodies-buffers/immunology-reagents/anti-non-human-primate-antibodies/cell-surface-antigens/apc-cy7-mouse-anti-human-cd3-sp34-2/p/557757
https://www.biolegend.com/en-us/products/apc-cyanine7-anti-human-cd4-antibody-3658?GroupID=GROUP28
https://www.bdbiosciences.com/us/reagents/research/antibodies-buffers/immunology-reagents/anti-non-human-primate-antibodies/cell-surface-antigens/apc-cy7-mouse-anti-human-cd8-rpa-t8/p/557760
https://www.bdbiosciences.com/us/applications/research/stem-cell-research/hematopoietic-stem-cell-markers/human/negative-markers/apc-h7-mouse-anti-human-cd14-m5e2/p/561384
https://www.biolegend.com/en-us/products/percp-cyanine5-5-anti-human-cd19-antibody-4226?GroupID=GROUP28
https://www.bdbiosciences.com/us/applications/research/b-cell-research/immunoglobulins/human/bv605-mouse-anti-human-igg-g18-145/p/563246
https://www.biolegend.com/en-us/products/pe-anti-human-igm-antibody-2878?GroupID=GROUP28
https://www.jacksonimmuno.com/catalog/products/109-035-006

Eukaryotic cell lines

Policy information about cell lines

Cell line source(s)	HEK293F cell line was obtained from ThermoFisher; HEK293T cell line was obtained from ATCC; HeLa-ACE2 was made in house from the HeLa cell line obtained from ATCC.
Authentication	No authentication was performed.
Mycoplasma contamination	The cell lines were not tested for mycoplasma.
Commonly misidentified lines (See ICLAC register)	No commonly misidentified cell lines were used.

Human research participants

Policy information about studies involving human research participants

Population characteristics	See supplementary Table S1.
Recruitment	Plasma and PBMCs from convalescent COVID patients were provided through the "Collection of Biospecimens from Persons Under Investigation for 2019-Novel Coronavirus Infection to Understand Viral Shedding and Immune Response Study" UCSD IRB# 200236. Plasma from pre-pandemic donors were provided by Primary Infection Resource Consortium (PIRC) UCSD IRB# 140093 and 191008. COVID patient samples were collected based on COVID-19 diagnosis regardless of gender, race, ethnicity, disease severity, or other medical conditions. The age and the ethnicity variables were relatively evenly distributed across the two human cohorts (COVID and pre-pandemic samples). The gender distribution in the pre-pandemic cohort could not be controlled due to the unavailability of the samples from female donors. The gender for individuals in the COVID cohort was evenly distributed.
Ethics oversight	Protocol was approved by the UCSD Human Research Protection Program.

Note that full information on the approval of the study protocol must also be provided in the manuscript.

Flow Cytometry

Plots

Confirm that:

- [x] The axis labels state the marker and fluorochrome used (e.g. CD4-FITC).
- [x] The axis scales are clearly visible. Include numbers along axes only for bottom left plot of group (a 'group' is an analysis of identical markers).
- [x] All plots are contour plots with outliers or pseudocolor plots.
- [x] A numerical value for number of cells or percentage (with statistics) is provided.

Methodology

Sample preparation	Frozen human PBMCs were re-suspended in 10 ml RPMI 1640 medium with 50% fetal bovine serum (FBS). After centrifugation at 400xg for 5 minutes, the cells were resuspended in a 5 ml FACS buffer (PBS, 2% FBS, 2mM EDTA). The cells were incubated with the mixture of fluorescently labeled antibodies to cell surface markers for 15 minutes on ice in the dark. The spike proteins of SARS-CoV-2,HKU1, and NL63 were conjugated to fluorescently labeled streptavidin, and each spike-probe was added to the Ab-cell mixture and incubated for 30 minutes on ice in the dark. FVS510 Live/Dead stain in the FACS buffer (1:300) was added to the cells and incubated on ice in the dark for 15 minutes. The stained cells were washed with FACS buffer and re-suspended in 500 µl of FACS buffer/10-20 million cells, passed through a 70 µm mesh cap FACS tube and ready for sort.

Instrument	Beckman Coulter Astrios sorter was used for flow cytometry data collection and single-cell sorting.
Software	FlowJo v10.7 was used for flow cytometry analysis.
Cell population abundance	Purity checks on sorted cells were not performed due to limitation of post-sort cell number.
Gating strategy	After the gating of lymphocytes (SSC-A vs. FSC-A) and singlets (FSC-H vs. FSC-A), live cells were identified by the negative FVS510 Live/Dead staining phenotype, then antigen-specific memory B cells were distinguished with sequential gating and defined as CD3-, CD4-, CD8-, CD14-, CD19+, IgM-and IgG+. Subsequently, spike-specific B cells were identified with the phenotype of AF488+BV421+ (CoV2/HKU1 double positive) or AF488+AF647+ (CoV2/NL63 double positive).

☒ Tick this box to confirm that a figure exemplifying the gating strategy is provided in the Supplementary Information.
